# The gut microbiota metabolite capsiate regulate SLC2A1 expression by targeting HIF‐1α to inhibit knee osteoarthritis‐induced ferroptosis

**DOI:** 10.1111/acel.13807

**Published:** 2023-03-08

**Authors:** Zhiyuan Guan, Xiao Jin, Zhiqiang Guan, Shengfu Liu, Kun Tao, Liying Luo

**Affiliations:** ^1^ Department of Orthopedics The Shanghai Tenth People's Hospital of Tongji University Shanghai China; ^2^ Department of Ophthalmology, Tongren Hospital Shanghai Jiao Tong University School of Medicine Shanghai China; ^3^ Department of Rheumatology and Immunology The First People's Hospital of Xuzhou Jiangsu Xuzhou China; ^4^ Department of Dermatology Xuzhou Municipal Hospital Affiliated with Xuzhou Medical University Jiangsu Xuzhou China

**Keywords:** Ferroptosis, HIF‐1α, iron homeostasis, osteoarthritis, SLC2A1

## Abstract

Ferroptosis is an iron‐dependent cell death that has been found to aggravate the progression of osteoarthritis (OA) and gut microbiota‐ OA axis refers to the bidirectional information network between the gut microbiota and OA, which may provide a new way to protect the OA. However, the role of gut microbiota‐derived metabolites in ferroptosis‐relative osteoarthritis remains unclear. The objective of this study was to analyze the protective effect of gut microbiota and its metabolite capsiate (CAT) on ferroptosis‐relative osteoarthritis in vivo and in vitro experiments. From June 2021 to February 2022, 78 patients were evaluated retrospectively and divided into two groups: The health group (*n* = 39) and the OA group (*n* = 40). Iron and oxidative stress indicators were determined in peripheral blood samples. And then in vivo and in vitro experiments, a surgically destabilized medial meniscus (DMM) mice model was established and treated with CAT or Ferric Inhibitor‐1 (Fer‐1). Solute Carrier Family 2 Member 1 (SLC2A1) short hairpin RNA (shRNA) was utilized to inhibit SLC2A1 expression. Serum iron was increased significantly but total iron binding capacity was decreased significantly in OA patients than healthy people (*p* < 0.0001). The least absolute shrinkage and selection operator clinical prediction model suggested that serum iron, total iron binding capacity, transferrin, and superoxide dismutase were all independent predictors of OA (*p* < 0.001). Bioinformatics results suggested that SLC2A1, Metastasis‐Associated Lung Adenocarcinoma Transcript 1 (MALAT1), and HIF‐1α (Hypoxia Inducible Factor 1 Alpha)‐related oxidative stress signaling pathways play an important role in iron homeostasis and OA. In addition, gut microbiota 16s RNA sequencing and untargeted metabolomics were used to find that gut microbiota metabolites CAT in mice with osteoarthritis were negatively correlated with Osteoarthritis Research Society International (OARSI) scores for chondrogenic degeneration (*p* = 0.0017). Moreover, CAT reduced ferroptosis‐dependent osteoarthritis in vivo and in vitro. However, the protective effect of CAT against ferroptosis‐dependent osteoarthritis could be eliminated by silencing SLC2A1. SLC2A1 was upregulated but reduced the SLC2A1 and HIF‐1α levels in the DMM group. HIF‐1α, MALAT1, and apoptosis levels were increased after SLC2A1 knockout in chondrocyte cells (*p* = 0.0017). Finally, downregulation of SLC2A1 expression by Adeno‐associated Virus (AAV) ‐SLC2A1 shRNA improves osteoarthritis in vivo. Our findings indicated that CAT inhibited HIF‐1a expression and reduced ferroptosis‐relative osteoarthritis progression by activating SLC2A1.

## Graphical abstract

Our findings indicated that CAT inhibited HIF‐1a expression and reduced ferroptosis‐dependent osteoarthritis progression by activating SLC2A1.

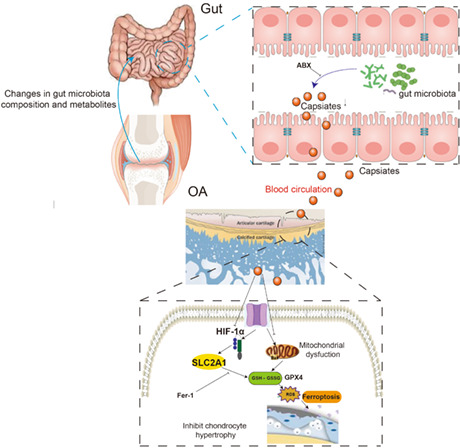

**Abbreviations**
AAV: Adeno associated Virus
ABX: antibiotics
BV/TV: bone volume over total volume
CAT: capsiate
CCK‐8: Cell Counting Kit‐8
CDKN1A: Cyclin‐Dependent Kinase Inhibitor 1A
DMM: destabilized medial meniscus
ECM: extracellular matrix
Fer‐1: Ferric Inhibitor‐1
GBD: Global Burden of Disease
GM: gut microbiota
GPX4: Glutathione Peroxidase 4
GSHPx: glutathione peroxidase
HIF‐1α: Hypoxia Inducible Factor 1 Alpha
MALAT1: Metastasis‐Associated Lung Adenocarcinoma Transcript 1
MCL: medial collateral ligament
MDA: malondialdehyde
MUC1: transmembrane glycoprotein mucin 1
OA: osteoarthritis
shRNA: short hairpin RNA
SLC2A1: Solute Carrier Family 2 Member 1
SOD: superoxide dismutase
Tb.Th: trabecular thickness
Tb.N: trabecular number
Tb.Sp: trabecular spacing

## 1 INTRODUCTION

Osteoarthritis (OA) is a chronic joint disease that gravely jeopardizes the health of the advanced ages (Barnett, 2018; Glyn‐Jones et al., 2015). According to a Global Burden of Disease (GBD) current dataset, the crude incidence rate of OA increased by 102% in 2017 compared to 1990 (Quicke et al., 2022). The risk factors are complex and multifactorial, and there are complicated interactions between OA and risk variables (age, gender, obesity, genetics, food, injury, malalignment, and aberrant joint loading) (Palazzo et al., 2016). In addition, increased catabolism in the extracellular matrix (ECM) of the articular cartilage induced by aging is a key factor in the development and progression of OA (Hamerman, 1993; Loeser et al., 2016). The confluence of many causes, such as cellular senescence, ferroptosis, inflammatory agents, degenerative joint lesions, the gut‐OA axis, and immunological response, may contribute to the pathogenesis of OA (Ebell, 2018; Glyn‐Jones et al., 2015; Guan et al., 2020; Molnar et al., 2021; Wang et al., 2020), all of which are associated with aging and tend to maximize through aging (Rahmati et al., 2017).

The gut microbiota (GM) plays an important role in maintaining host physiological homeostasis and health, including nutrient assimilation, toxin elimination, maintenance of immune homeostasis, and hormone delivery (Andrews & Vasanthakumar, 2023; Bauer‐Estrada et al., 2023; Procházková et al., 2023). GM is closely related to a variety of diseases including trauma repair, osteoporosis, and Parkinson's disease (Hu et al., 2016; Liu, Chen, et al., 2021; Zmora et al., 2019). In our previous studies, we found that gut microbiota depletion caused by antibiotics can alleviate the progression of OA (Guan et al., 2020; Guan, Luo, et al., 2022). Changes in the abundance of certain specific gut microbiota such as Desulfovibrionales and Ruminiclostridium often lead to the progression of osteoarthritis (Yu et al., 2021). Intestinal flora and metabolites may influence the course of osteoarthritis by regulating the intestinal mucosal barrier, intestinal metabolites, and immune levels, among other perspectives (Liu, Tian, et al., 2022). In our study, fecal 16S rRNA sequencing and metabolomics were used to explore the changes in gut microbiota‐derived metabolites in osteoarthritis, and then capsiate (CAT) was regarded as the gut microbiota metabolites which was drastically decreased in OA. CAT a nonpungent capsaicin analog, and its dihydroderivative dihydrocapsiate are the major capsaicinoids of the nonpungent red pepper cultivar CH‐19 Sweet (Deng et al., 2021; Grgic et al., 2022; Gupta et al., 2022). CAT has been found to promote energy expenditure and metabolism, inhibit fat accumulation, antioxidant, anti‐inflammatory, and anti‐tumor properties (Grgic et al., 2022; Ludy et al., 2012; Zang et al., 2018). Gut microbiota metabolite capsiate can activate ferroptosis in intestinal ischemia reperfusion (Deng et al., 2021). The role and mechanism of CAT in osteoarthritis are unknown.

Iron is one of the essential elements for humans, and has been implicated in various human diseases, including hereditary hemochromatosis, hemophilia, thalassemia, sickle cell disease, aging, estrogen shortage, and OA (Cai et al., 2021; Sun et al., 2021). Iron overload also may exacerbate the progression of osteoarthritis (Burton et al., 2020). Furthermore, there was a closed relationship between iron consumption and knee OA development, for example with an inflection point at 16.5 mg/day and little risk from 10.9 to 23.3 mg/day of iron intake (Wu et al., 2022). However, there are currently no studies to investigate the relationship between iron hemostasis and OA and its possible mechanism.

Ferroptosis is a ferrous ion‐dependent, cell death mode triggered by lipid peroxidation and reactive oxygen species accumulation, and plays a significant role in the course of many diseases (Jiang et al., 2021; Tang et al., 2021), and we previously recognized the important role of ferroptosis in sarcoma (Guan, Liu, et al., 2022). In addition, ferroptosis also plays an essential role in OA (Miao et al., 2022; Yang et al., 2021; Yao et al., 2021; Zhou et al., 2021). Previous studies found that ferroptosis inhibitor increases the expression of matrix metallopeptidase 13(MMP‐13) while decreasing collagen II expressions in chondrocytes. Furthermore, the level of ferroptosis could be inhibited by intraarticular injection of ferroptosis inhibitor (Yao et al., 2021). Glutathione Peroxidase 4(GPX4), which considers key target genes of ferroptosis, downregulation could increase chondrocyte sensitivity to oxidative stress and aggravate extracellular matrix degradation via the mitogen‐activated protein kinase pathway, demonstrating that ferroptosis contributes to the pathogenesis of OA (Miao et al., 2022). Besides, the relationship between Iron and ferroptosis is currently unknown (Doll & Conrad, 2017). In addition, gut microbiota and related metabolites also activate ferroptosis, which plays an important role in a variety of diseases (Deng et al., 2021; Liu, Gao, et al., 2022). There is growing evidence that cells undergoing ferroptosis can activate factors of the innate immune system that lead to lipid peroxidation, the underlying cause of tissue damage and organ failure (Zhou et al., 2020). Understanding the changes and molecular biological signaling pathways of ferroptosis in OA may represent valuable therapeutic targets for OA treatment.

Therefore, this study aimed to investigate changes in gut microbiota and metabolites in osteoarthritis and found the role of CAT in osteoarthritis which prevents ferroptosis. In addition, we further investigated the possibile mechanism of HIF‐1a expression and SLC2A1 which effect by gut microbiota metabolite CAT and provide a theoretical basis for CAT treatment of osteoarthritis.

## 2 MATERIALS AND METHODS

### 2.1 Ethics approval

This is a single‐center, cross‐sectional study. This study was authorized by our hospital's ethical committee as well as the institutional review board (IRB) (SHSY:6420‐1152). All patients were given information and asked to sign an informed consent form. These studies were performed according to the CONSORT standards and complied with the Helsinki Declaration. All experimental protocols and animal handling procedures were conducted according to the recommendations in the Guide for the Care and Use of Laboratory Animals, published by the National Institutes of Health (Publications No. 80–23, revised in 1996). This study was approved by the Experimental Animal Committee of our hospital (SHSY:6420‐1222).

### 2.2 Patients

In June 2021 and February 2022, 78 patients were recruited from our hospital at the authors' institution. There were 38 patients in the control group and 40 patients in the OA group. The patients included in this study were at least grade 2 evaluated by the Kellgren and Lawrence method for categorization of knee OA and supra‐patellar bursitis. (1) The thickness of the supra‐patellar bursa was measured to be >2 mm, as validated by musculoskeletal ultrasonography. (2) Patients had a history of knee joint steroid injections, physical modalities therapies, and oral Nonsteroidal Antiinflammatory Drugs (NSAIDs), but there was no discernible reduction in knee discomfort. (3) Patients had a history of knee joint steroid injections, physical modalities therapies, and oral NSAIDs, but there was no discernible reduction in knee discomfort. (4) Musculoskeletal ultrasonography revealed increased hypoechoic changes in the pes anserinus complex, indicating inflammation of the tendons and bursitis. (5) Knee OA was the primary cause of supra‐patellar bursitis, protruded meniscus with bulging medial collateral ligament (MCL), and pes anserine inflammation, not viral or inflammatory knee diseases. Before taking part in this study, they completed an informed consent form. Patient demographics and other variables, such as age, gender, location, time of operation, smoking status, hypertension, and other cardiac problems, were documented.

### 2.3 Clinical indicators

In a subset of 78 patients, serum concentrations of albumin (LabCorp), hemoglobin (HB) (Covance), pro‐albumin (Covance), superoxide dismutase, monoamine oxidase (MAO), glutathione reductase, total iron binding capacity, transferrin, e‐transferrin receptor, and serum iron (Covance) were examined. The concentrations were calculated using the standard method of calculating areas under the peaks.

### 2.4 Prognostic nomogram analysis

To decrease multivariate data and select risk variables for osteoporosis at rotator cuff rupture, the LASSO (Least Absolute Shrinkage and Selection Operator) approach was applied. Non‐zero LASSO regression coefficients were used in the training set. To build a prediction model, multiple logistic regression analysis was done on chosen variables in the LASSO regression model. To analyze the calibration of the non‐adhesive coating nomogram, a calibration curve was produced, and the Harrell c index was calculated to quantify the discriminative strength of the non‐adhesive coating nomogram. To assess net benefit, and decide whether to do decision curve analysis to find clinically meaningful non‐compliance nomograms. The diagnostic effectiveness of the clinical factor model was evaluated using the training and validation toolkit's suitable ROC curve (AUC) (Balachandran et al., 2015; Huang et al., 2016; Kidd et al., 2018; Vickers et al., 2008; Xing et al., 2017).

### 2.5 Data analysis

Gene Expression Omnibus (GEO) was used to acquire clinical information on DR patients. We obtained the dataset GSE98918 from the GEO. Agilent microarrays were used to identify gene transcripts that were differentially expressed in meniscus tissues obtained from 12 patients with osteoarthritis and 12 patients without osteoarthritis (arthroscopic partial meniscectomy), so patient consent and ethics committee approval were unnecessary. GEO2R, an online analytic tool was used (Barrett et al., 2013).

DAVID 6.8, Metascape, and WebGestalt were used to perform functional enrichment analysis on DEGs. The Funrich enrichment analysis method was also used to examine miRNA biochemical pathways. The difference was statistically significant at *p* < 0.05. TRING, an online database capable of obtaining the connections between a collection of proteins, was employed in the study of the PPI network (Szklarczyk et al., 2017) to predict protein–protein interactions (PPIs). Furthermore, the PPI network was created and displayed using the Cytoscape v3.6.0 software. MCODE's job is to choose the critical sub‐networks, i.e., modules. A PPI module is a group of PPI modules, each with its own purpose.

### 2.6 Gene–miRNA interaction networks and miRNA‐lncRNA prediction

In addition to the analytical tools indicated above, we utilized miRWalk 2.0 (http://mirwalk.umm.uni‐heidelberg.de/) and StarBase v2.0 (https://starbase.sysu.edu.cn/) to determine targeted key miRNAs and lncRNA and establish gene–miRNA and miRNA‐lncRNA interaction networks. As evidence of the importance of lncRNAs, we chose cross‐linked diagrams illustrating the interaction of each miRNA's computed findings.

### 2.7 Experimental post‐traumatic OA in mice

Mice were housed in a standard animal facility under a controlled temperature (22°C) and photoperiod (12‐h light and 12‐h dark) and had free access to fresh water and food.

To observe the role of ferroptosis in osteoarthritis, mice were randomly divided into four groups. (1) sham‐operated group (*N* = 9): the same procedure as the destabilization of the medial meniscus (DMM) except that no medial meniscal instability was performed; (2) sham + Ferric Inhibitor‐1 (Fer‐1) (*N* = 9): mice were injected intraperitoneally with 10 mg/kg ferroptosis inhibitor Ferric Growth Inhibitor‐1 (Fer) (MedChemExpress) for 4 h; (3) OA group (DMM surgery) (*N* = 9); (4) OA + Fer‐1 (*N* = 9): the mice were intraperitoneally injected with 10 mg/kg ferroptosis inhibitor Fer‐1. Then the mice were treated with antibiotics(ABX) (vancomycin, 100 mg/kg; neomycin sulfate 200 mg/kg; metronidazole 200 mg/kg; and ampicillin 200 mg/kg) intragastrically once each day for 1 week to deplete the gut microbiota. Mice were given an intraperitoneally injection of Fer‐1, ABX, or vehicle using 33‐gauge needles (Hamilton Company) and 10 μL CASTIGHT syringes (Hamilton Company). The injection was repeated twice a week for 8 consecutive weeks (*N* = 9 in each group).

To explore the protective effect of CAT on OA in vivo, the mice were randomly divided into 4 groups (1) Sham group (*N* = 9); (2) Sham + CAT group (*N* = 9): the mice were intraperitoneally injected with 2 mg/kg CAT (Alomone Labs, Shanghai, China) for 4 h; (3) OA group (*N* = 9); (4) OA+ CAT group (*N* = 9): the mice were intraperitoneally injected with 2 mg/kg CAT. Pentobarbital (50 mg/kg, intraperitoneal injection) was used to kill the mouse involved in this investigation. The techniques and findings were published in accordance with the standards of Animal Research Reporting in Vivo Experiments, and DMM was generated by transecting the medial menisco‐tibial ligament in the right knee, as described by Dr. Glassion (Glasson et al., 2005, 2007). The mice were euthanized 8 weeks later, and the knee joints were collected and preserved in 4% paraformaldehyde for future research.

SLC2A1 short hairpin RNA (shRNA) fragment was cloned into adeno‐associated virus (AAV) vector GV478 (U6‐MCS‐CAG‐EGFP) (Shanghai Genechem Co., Ltd) to construct AAV‐shSLC2A1. AAV‐293 cells were co‐transfected with recombinant AAV‐shSLC2A1, pAAV‐RC, and pHelper for AAV packaging. AAV was collected from the AAV‐293 cell supernatant, and then condensed and purified. Eight‐week‐old C57BL/6 mice were anesthetized with pentobarbital, and the skin above the articular joint was shaved. Mice were injected intraarticularly with 10 mL of either 1.0 * 10^10^ v.g AAV‐NC, AAV‐shSLC2A1, or CAT (0.1 mg/kg) using 33‐gauge needles (Hamilton Company) and 10 μL CASTIGHT syringes (Hamilton Company), as shown in the previous studies (Miao et al., 2022) (*N* = 9 in each group).

### 2.8 Micro‐CT analysis

The micro‐CT (Inveon, Siemens, Erlangen, Germany) has a spatial resolution of 55 kVp and 145 mA, an integration time of 300 ms, and a 6‐mm isotropic voxel edge. The medial tibial plateau, which is defined as the area of the tibia superior to the growth plate, posterior to the insertion of the anterior cruciate ligament, and medial to the midline of the intercondylar notch, was included in the study. We examined subchondral bone in this location using morphometric assessments of bone volume over total volume (BV/TV), trabecular thickness (Tb.Th), trabecular number (Tb.N), and trabecular spacing (Tb.Sp) in the medial tibial subchondral trabecular bone, as previously described (Guan et al., 2020; Longobardi et al., 2017).

### 2.9 Histology assay

The fixed knees were decalcified in 10% ethylenediaminetetraacetic acid (EDTA) for 14 days before being embedded in paraffin and sliced into 5 m frontal slices. Images were taken using a stereomicroscope after staining with Safranin‐O/FastGreen, immunofluorescence, scanning electron microscope, and HE staining (SMZ745T, Nikon, Tokyo, Japan). Anti‐MMP‐13 antibodies (ab39012, Abcam) were used to detect protein expression in the knee tissue. The severity of OA in cartilage was determined using the Osteoarthritis Research Society International histology score approach (OARSI) (Glasson et al., 2010).

### 2.10 Measurement of GSH, MDA, and H_2_O_2_

Anesthetized mice were decapitated and their eyes were immediately enucleated. Lenses were removed from enucleated eyes due to their high GSH concentration. Lenseless eyes were homogenized in pre‐chilled 0.2 m potassium phosphate buffer, pH 7.0. We measured malondialdehyde (MDA) by thiobarbituric acid method, measured superoxide dismutase (SOD) by the xanthine oxidase method, conducted glutathione peroxidase (GSH‐Px) by NADPH method and determined catalase (CAT) by coloration method in the retinal tissue homogenates using commercial kits (Beyotime Institute of Biotechnology, Shanghai, China) according to the previous studies (Lawrence et al., 1978; Lowry et al., 1951; Reed et al., 1980; Richard et al., 1992; Romero et al., 1998). H_2_O_2_ was determined using commercial kits (Beyotime Institute of Biotechnology, Shanghai, China) according to the manufacturer's procedures in cartilage tissue sample homogenates (Arunachalam et al., 2021).

### 2.11 Isolation and culture of chondrocytes cells

Chondrocytes were extracted and cultivated from 5‐day‐old C57BL/6J mice as previously described (Sun et al., 2019). The cartilage in the knee joints was removed and dissected into bits. The cartilage was processed for 30 min with 0.25% trypsin and 6 h with 0.25% type 2 collagenase. The primary chondrocytes were resuspended and cultivated at 37°C in a humidified environment of 5% CO_2_ in DMEM/F12 media containing 10% fetal bovine serum, 1% penicillin, and 1% streptomycin sulfate. The trials employed chondrocytes from the first and second passages (Guo et al., 2022).

### 2.12 SLC2A1 knockout chondrocytes cells

Gima (Guangzhou, China) chemically produced specific small interfering RNA (siRNA) targeting the mouse SLC1A3 gene and transfected it into cells using Lipofectamine 3000 transfection reagent according to the manufacturer's instructions (Thermo Fisher, UT, USA). The SLC2A1 siRNA sense strand sequence was previously disclosed (Fullana et al., 2019).

### 2.13 Cell viability assays

The Cell Counting Kit‐8 (CCK‐8) (Boster, China, AR1160) test was used to determine chondrocyte vitality. Chondrocytes were plated at a density of 3000 cells per well in 96‐well plates with five duplicate wells. After adhesion for 24 h, the cells were treated for 24 h with DFO at various doses alone or in combination with IL‐1 or erastin. After removing the medium, 10% CCK‐8 solution was added to each well and incubated at 37°C away from light for 1 h. A microplate reader was used to measure the absorbance at 450 nm (Thermo Fisher Scientific, Vantaa, Finland).

### 2.14 qPCR analysis

Chondrocytes were stripped of muscle and connective tissue before being flash‐frozen in liquid nitrogen and kept at 80°C. A bessman tissue pulverizer was used to smash frozen chondrocytes under liquid nitrogen conditions (Spectrum Laboratories, Rancho Dominguez, CA, USA). Trizol reagent was used to extract total RNA (Invitrogen, Carlsbad, CA, USA). The levels of expression of bone metabolisms, glucose metabolism, and inflammation‐related genes, such as Endothelial PAS Domain Protein 1 (EPAS1), Transmenbrane glycoprotein mucin1 (MUC1), CD44 Molecule (CD44), SLC2A1, Cyclin‐Dependent Kinase Inhibitor 1A (CDKN1A), and Dual Specificity Phosphatase 1 (DUSP1), have been uploaded in Table S1. With the Sham group as the baseline, the mRNA of on‐related genes such as EPAS1, MUC1, CD44, SLC2A1, CDKN1A, and DUSP1 is computed (Zhou et al., 2022).

### 2.15 16S rRNA gene sequencing

After DNA extraction, the V4 region of the 16S rRNA gene was amplified using specific barcode primers (V4F, 5′‐GTGTGYCAGCMGCCGCG GTAA‐3′, and V4R, 5′‐CCGGACTACNVGGG TWTCTAAT‐3′). The PCR amplification products were mixed in equal amounts measured by QuantiFluor. All samples were subjected to paired‐end sequencing on the Illumina Hiseq PE250 (San Diego, CA, USA) platform. High‐throughput sequencing analysis of bacterial rRNA genes was processed using the Quantitative Insights into Microbial Ecology (QIIME, version 1.9.1) software suite. The calculated p‐value was gone through FDR Correction, taking FDR ≤0.05 as a threshold (Deng et al., 2021).

### 2.16 Fecal metabolic profiling

The nontargeted metabolomics procedure was performed by ESI‐QTOF/MS (Xevo 121 G2‐S Q‐TOF, Waters) and UPLC‐QTOF/MS (ACQUITY UPLC I‐Class, Waters) as described previously. Targeted metabolomics (capsiate measurement) was performed by LC–MS/MS. The procedure included integration, normalization, and peak intensity alignment. In the positive data set, a list of m/z and retention time with corresponding intensities was provided for all metabolites in every sample. Then, the processed data set was then entered into the SIMCA‐P software package (v13.0, Umetric, Umea, Sweden). The normalized data were then used to perform principal component analysis (PCA) and orthogonal to partial least squares‐discriminate analysis (OPLS‐DA) with VIP > 1 as a threshold. The chromatographic separation was performed on the Thermo Scientific Prelude SPLC system, and detection was performed on the Thermo TSQ Vantage triple quadrupole mass spectrometer (Deng et al., 2021).

### 2.17 Molecular modeling and docking of SLC2A1 and HIF‐1α

As shown previously described, the docking study of SLC2A1 and HIF‐1α was performed. Briefly, the crystal structure of SLC2A1 was aligned to the crystal structure of HIF‐1α by ChEMBL database (https://www.ebi.ac.uk/chembl/#). And then using CB‐Dock software (http://clab.labshare.cn/cb‐dock/php/index.php) to perform the docking study and the docking results were visualized using CB‐Dock software (Yang et al., 2022).

### 2.18 Western blotting

Chondrocytes were planted at a density of 5 × 10^5^ cells per well in six‐well plates and adhered for 48 h. Cells from various treatment groups were extracted for 30 min on ice using RIPA lysis buffer (Boster, China, AR0102) containing 1% phenylmethylsulfonyl fluoride and 1% phosphatase inhibitor cocktail. The extract was collected and centrifuged for 30 min at 12,000 ***g*** and 4°C. The supernatant was then collected, and the protein concentrations in each sample were determined using a BCA assay kit (Boster, China, AR0146). Protein samples were separated using SDS‐PAGE and transferred to PVDF membranes (Millipore, USA). After blocking with 5% skim milk for 1 h at room temperature, the membranes were incubated overnight at 4°C with specified primary antibodies, followed by 1 h at room temperature with secondary antibodies. Protein bands were seen using a chemiluminescence reagent (Boster, China), and photos were captured using a Bio‐Rad scanner (Hercules, CA, USA) (Yao et al., 2021).

### 2.19 Statistical analysis

All measures are given as mean ± standard deviation (SD), with a *p*‐value of 0.05 considered statistically significant. GraphPad Prism 8.02 (La Jolla, California, USA) and one‐way ANOVA were used to analyze the data, followed by Tukey's multiple test. All experimental replicates were repeated three times.

## 3 RESULTS

### 3.1 The role of iron overload in osteoarthritis patients

Table 1 shows the summary of demographic and clinical characteristics, as well as the variations in these parameters between OA and control participants. In the OA group, 55.26% of the participants are female, while 55% are female in the control group. The patients in the OA group and the control group are 53.26 ± 3.65 and 54.36 ± 7.25 years old, respectively.

**TABLE 1:** Demographic and clinical characteristics.

Characteristics	Normal (*N* = 38)	OA (*N* = 40)	All group (*N* = 78)	*p* Value
Female, *n* (%)	21 (55.26)	22 (55.00)	43 (55.12)	0.36
Age (y)	53.26 ± 3.65	54.36 ± 7.25	53.69 ± 4.58	0.15
BMI (cm^2^/kg)	23.65 ± 3.62	24.62 ± 3.65	23.95 ± 3.95	0.85
Weight (kg)	66.86 ± 3.62	67.85 ± 4.26	66.91 ± 4.28	0.54
Height (cm)	165.96 ± 4.69	165.94 ± 7.59	165.29 ± 9.56	0.43
Diabetes, *n* (%)	3 (7.89)	4 (10.00)	7 (8.79)	0.32
*Hypertension, n (%)*				
0	21 (55.36)	22 (55.00)	43 (55.12)	0.32
1	11 (28.94)	10 (25.00)	21 (26.92)	0.18
2	4 (10.52)	7 (17.50)	11 (14.10)	0.11
3	2 (0.53)	1 (2.50)	3 (3.84)	0.36
Other heart disease, *n* (%)	14 (36.84)	15 (37.50)	29 (36.50)	0.52
Stoke, *n* (%)	2 (7.89)	4 (10.00)	4 (3.8)	**0.04**

Bold indicates statistical significant value(p<0.05).

**FIGURE 1:**
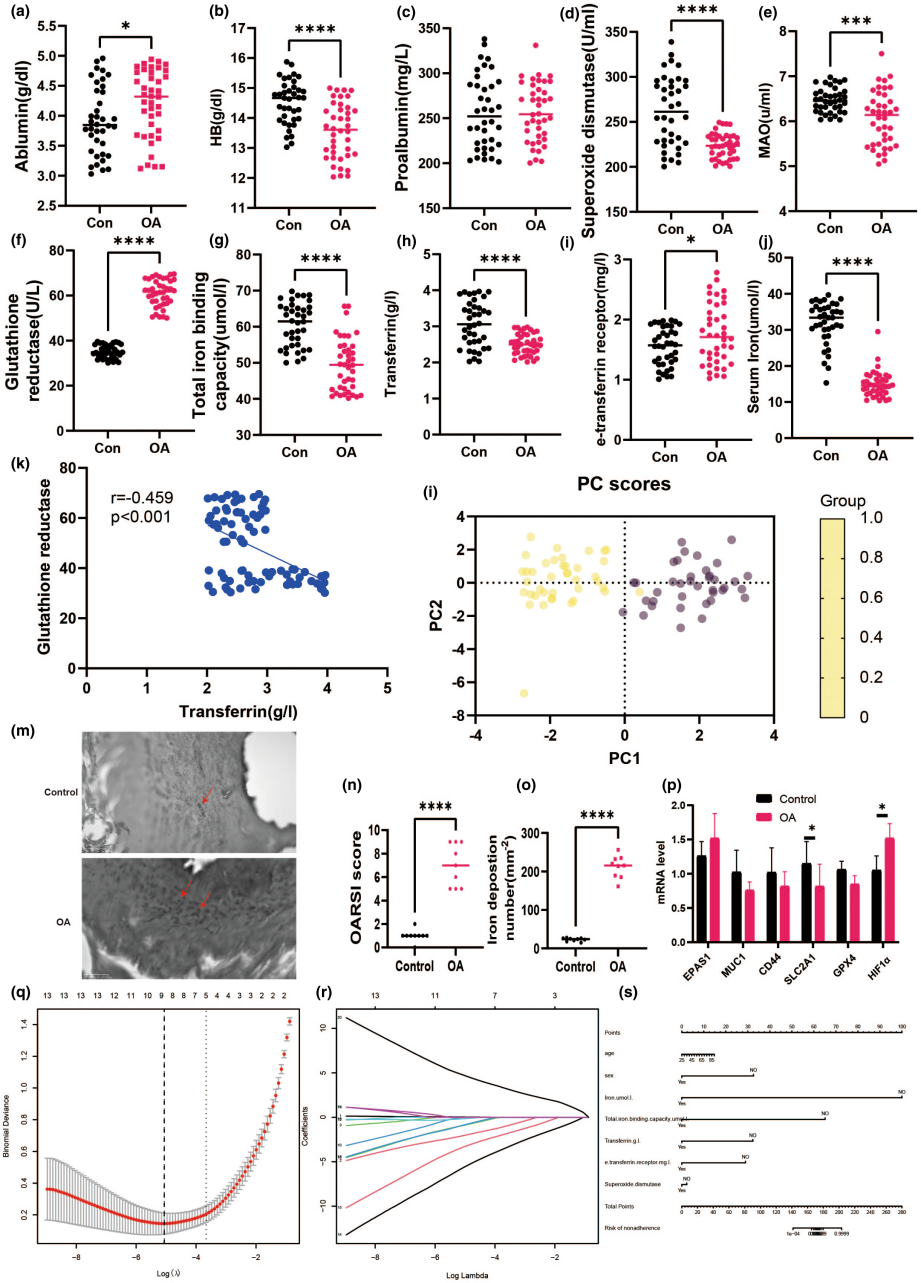
Iron homeostasis‐related indicators and prognostic nomogram analysis in OA patients. (a) Serum iron. (b) HB, (c) proablumin. (d) Superoxide dismutase. (e) MAO. (f) Glutathione reductase. (g) Total iron binding capacity. (h) Transferrin. (i) e‐transferrin receptor. (J) serum iron. (k) correlation analysis between glutathione reductase and transferrin. (l) PCA analysis. (m) Distribution of expression of iron regulators in synovial tissue of healthy controls and osteoarthritis. (n) OARSI score. (o) iron deposition number. (p) mRNA level in synovial tissue. (q) Optimal parameter (lambda) selection in the LASSO model uses fivefold cross‐validation using minimal criteria. (r) LASSO coefficient profiles of the 22 characteristics. A coefficient profile plot was constructed against the log(lambda) sequence. Vertical line was drawn at the value determined using fivefold cross‐validation, where optimum lambda resulted in five features with nonzero coefficients. Developed nomogram. HB, hemoglobin; MAO, monoamine oxidase; OA, osteoarthritis; AUC, area under the curve; GSH, glutathione; GSH/GSSH, glutathione/oxidized glutathione; OARSI, Osteoarthritis Research Society International. *p < 0.05; **p < 0.01; ***p < 0.001.

Iron deficiency has a deleterious impact on joint homeostasis (Cai et al., 2021). As a result, the first set of questions concerns the relationship between iron burden and OA patients. Serum iron levels were higher in OA patients than in control patients, but transferrin and total iron binding capacity were lower than in the control group (Figure 1). Oxidative stress markers such as blood superoxide dismutase and MAO levels were considerably lower in OA patients but significantly higher in the control group. Figure 1k,l depicts the PCA analysis showing the relationship between glutathione reductase, and transferrin. In addition, the distribution of expression of iron regulators in synovial tissue is demonstrated in Figure 1m,n and the deposition number of iron ions in synovial tissue and OARSI score in cartilage were significantly increased in the osteoarthritis group compared to the normal group.

We discovered a substantial link between osteoarthritis and iron‐related markers using clinical data analysis. However, there is a scarcity of research on the bioinformatic elements of the genetic link between osteoarthritis and iron metabolism. As a result, we investigated the link between osteoarthritis and ferroptosis by clinical dataset. Figure S1 shows box plots, major cause analysis plots, UMAP plots, volcano plots, and heat maps obtained from the GEO database using GSE24265. We also obtained the Ferroptosis Database (FerrDb) dataset, which included 388 genes, and intersected it with GSE98918 to detect 34 ferroptosis DEGs (Table S2). And then, we initially imported the 34 intersected DEGs into WebGestalt, DAVID, Metascape (Table S3). Notably, it was found that biological processes were significantly enriched in response to the HIF‐1 signaling pathway, reaction to oxygen levels, and response to nutrition levels (Figure S1).

The PPI network (interaction score >0.4) was derived from a molecular network (Figure S2). Key module studies showed genes associated in the HIF‐1α pathways with the highest MCODE scores, including EPAS1, CA9, MUC1, CD44, SLC2A1, BNIP3, TLR4, CDKN1A, and TGFB1. Furthermore, using Metascape, the functional analysis for Cluster 1 revealed that these nine genes were mostly involved in the HIF‐1α signaling pathway, the control of reactive oxygen species metabolic processes, and type 2 papillary renal cell carcinoma (Table S4).

To verify the precision and trustworthiness of our findings, the crosslinked miRNAs were chosen using the miRWalk and miRTarBase databases. Table S5 displays the miRNAs with the greatest number of cross‐linked genes (>2). The biochemical pathways comprised estrogen receptor signaling in plasma, LKB1 signaling events, and VEGF signaling events (Figure S3). Following cross‐linking, one lncRNA targeting three critical miRNAs, namely MALAT1, was discovered (Figures S4‐S6). We speculated that SLC2A1 may be essential for HIF‐1α‐induced osteocyte ferroptosis. Therefore, mRNA level in cartilage was an analysis that SLC2A1 was decreased significantly but HIF‐1α was increased markedly than the control group. Taken together, these results suggest that there are associations between OA and iron homeostasis.

### 3.2 Prognostic nomogram analysis between iron and OA

**FIGURE 2:**
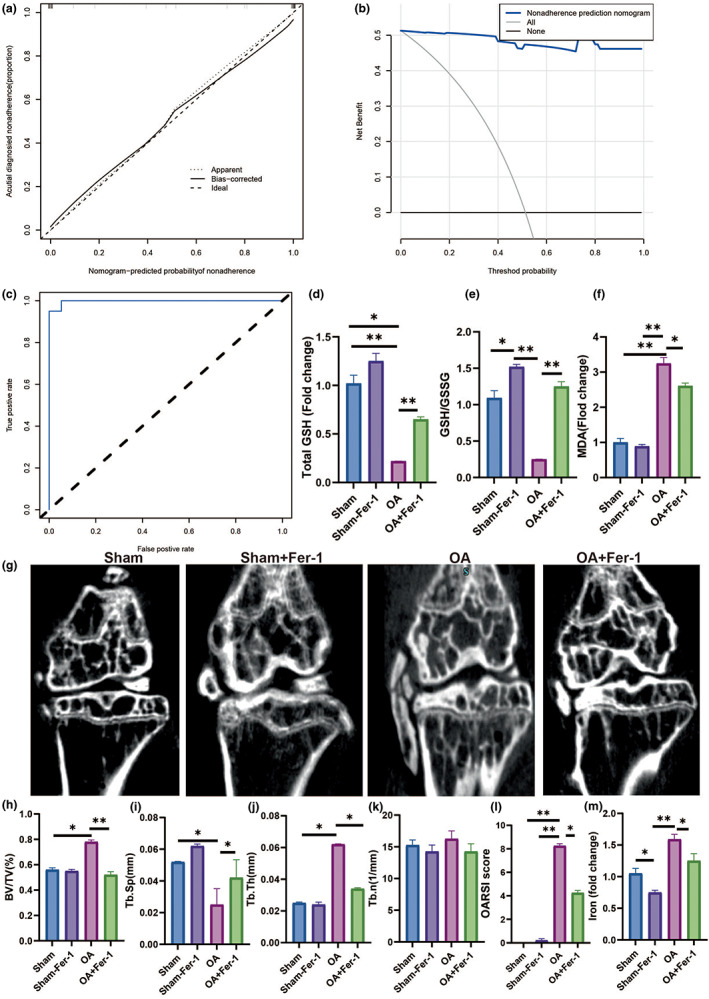
Ferropotisis play an important role in OA. (a) Calibration curves of the nonadherence nomogram prediction in the cohort. (b) Decision curve analysis for the nonadherence nomogram. (c) AUC curve in iron homeostasis in OA patients. (d) Total GSH. (e) GSH/GSSH. (f) MDA. (g) Representative figure of Micro‐CT. (h) BV/TV in tibia. (i) Tb.Th in tibia. (j) Tb.N in tibia. (k) Tb.Sp in tibia. (l) OARSI score in tibia. (m) Iron load in tibia. All data are from *n* = 3 independent experiments. OA, osteoarthritis; AUC, area under the curve; GSH, glutathione; GSH/GSSH, glutathione/oxidized glutathione; BV/TV, bone volume over total volume; Tb.Th, trabecular thickness; Tb.N, trabecular number; Tb.Sp, trabecular spacing; OARSI, Osteoarthritis Research Society International. *p < 0.05; **p < 0.01; ***p < 0.001.

As shown in Table 2, multiple linear regression models revealed a link between glutathione reductase and e‐transferrin receptor and OA. Gender and TIBC were also found to be independent predictors of OA in multivariate logistic regression models in Table 3. Based on 78 patients in the cohort (4:1 ratio; Figure 1q,r), 15 variables were reduced to five possible predictors with nonzero coefficients in the LASSO regression model. Sex, age, serum iron, TIBC, Transferrin, e‐transferrin receptor, and superoxide dismutase were among these characteristics (Table 4). The nomogram was created using the model that included the aforementioned independent predictors (Figure 1s). Meanwhile, in this cohort, the calibration curve of the nonadherence risk nomogram for risk prediction in OA patients revealed good consistency (Figure 2a). The C‐index for the prediction nomogram for the cohort was 0.997 (95 percent CI, 0.265–1.728) and was validated to be 0.902 after bootstrapping validation, indicating that the model had strong discrimination. Figure 2b depicts the decision curve analysis for the iron‐linked indicators nomogram, and the AUC curve indicates that the area under the curve was 0.978 (Figure 2c). In conclusion, Iron metabolism may be a valuable indicator for the prognosis of osteoarthritis.

**TABLE 2:** Multiple line regression of iron hemostasis and OA patients.

Variable	Estimate	Standard error	95% CI (asymptotic)	|*t*|	*p* Value
Intercept	0.9119	0.6183	−0.3277 to 2.152	1.475	0.1461
Age	−0.0006763	0.001932	−0.004550 to 0.003197	0.35	0.7277
Sex	−0.02022	0.06767	−0.1559 to 0.1154	0.2988	0.7662
Size	−0.01533	0.04687	−0.1093 to 0.07864	0.3271	0.7449
Weight	0.002693	0.00592	−0.009177 to 0.01456	0.4549	0.651
High	0.0000821	0.0007953	−0.001512 to 0.001677	0.1032	0.9182
BMI	−0.004674	0.01689	−0.03853 to 0.02918	0.2768	0.783
HP[2]	0.02134	0.06369	−0.1064 to 0.1490	0.335	0.7389
HP[1]	0.06273	0.05678	−0.05111 to 0.1766	1.105	0.2742
HP[3]	−0.1295	0.193	−0.5165 to 0.2575	0.6709	0.5051
LVEF	−0.0252	0.1278	−0.2815 to 0.2311	0.1972	0.8444
MI	−0.005147	0.0766	−0.1587 to 0.1484	0.0672	0.9467
DM	−0.009952	0.06367	−0.1376 to 0.1177	0.1563	0.8764
Ablumin (g/dL)	−0.02849	0.03907	−0.1068 to 0.04984	0.7292	0.4691
Hb (g/dL)	−0.04916	0.02744	−0.1042 to 0.005841	1.792	0.0787
Proalbumin	−0.0004952	0.000689	−0.001877 to 0.0008862	0.7187	0.4754
Iron (umol/L)	−0.01405	0.003729	−0.02153 to −0.006578	3.769	0.0004
Total iron binding capacity (umol/L)	−0.003797	0.003322	−0.01046 to 0.002862	1.143	0.258
Transferrin (g/L)	−0.06137	0.04733	−0.1563 to 0.03351	1.297	0.2002
e‐Transferrin receptor (mg/L)	−0.04825	0.05241	−0.1533 to 0.05683	0.9206	**0.0361**
Superoxide dismutase	−0.0006445	0.000719	−0.002086 to 0.0007969	0.8964	0.374
MAO	0.05606	0.04426	−0.03267 to 0.1448	1.267	0.2107
Glutathione reductase	0.02225	0.003088	0.01606 to 0.02844	7.205	**<0.0001**

Bold indicates statistical significant value(p<0.05).

**TABLE 3:** Multiple logistic regression analysis risk prediction model.

Intercept and variable	Prediction model				
	*β*	HR (95% CI)	2.50%	97.50%	*p* Value
Intercept	1.3161257	3.72E+00	9.07E−02	1.97E+02	0.491
Age	0.0212668	1.02E+00	9.65E−01	1.08E+00	0.4613
Sex	−2.011675	1.34E−01	1.78E−02	6.66E−01	0.0227
Iron	−21.71691	3.70E−10	2.29E−245	2.60E+30	0.9906
Total iron binding capacity	−2.823348	5.94E−02	5.34E−01	7.51E+01	0.032
Transferrin	−20.42405	1.35E−09	2.20E−03	5.45E−01	0.997
e‐Transferrin receptor	−1.28234	2.77E−01	2.21E−02	2.51E+00	0.261
Superoxide dismutase	−1.354333	2.58E−01	5.12E−02	1.67E+00	0.231
Glutathione reductase	22.881624	8.66E+09	1.06E−01	2.19E+00	0.996

### 3.3 The relationship between ferroptosis and osteoarthritis in mice

To verify the above hypothesis, we first analyzed the role of ferroptosis in the development of OA. Ferrostatin‐1 (Fer‐1) is a safe and effective iron‐chelating agent that has been licensed by the Food and Drug Administration for the treatment of iron overload diseases (Liu et al., 2020). As a result, we employed Fer‐1 to minimize ferroptosis and studied its effect on OA in mice (Figure S7). Total GSH, GSH/GSSH ratio, and MDA statistically decreased in the Fer‐1 + OA group than OA group (Figure 2d–f). We utilized Micro‐CT and pathological analysis to examine the microstructure of the subchondral bone in osteoarthritic mice, as seen in Figure 2g‐m. The Fer‐1 + OA group had a lower joint OARSI score and BV/TV but higher Tb.Sp compares with the OA group. Meanwhile, the Fer‐1 + OA group had a lower level of iron than the DMM group. In summary, inhibiting ferroptosis with Fer‐1 can improve the progress of osteoarthritis.

### 3.4 Changes in gut microbiota and their metabolites in mice with osteoarthritis

**FIGURE 3:**
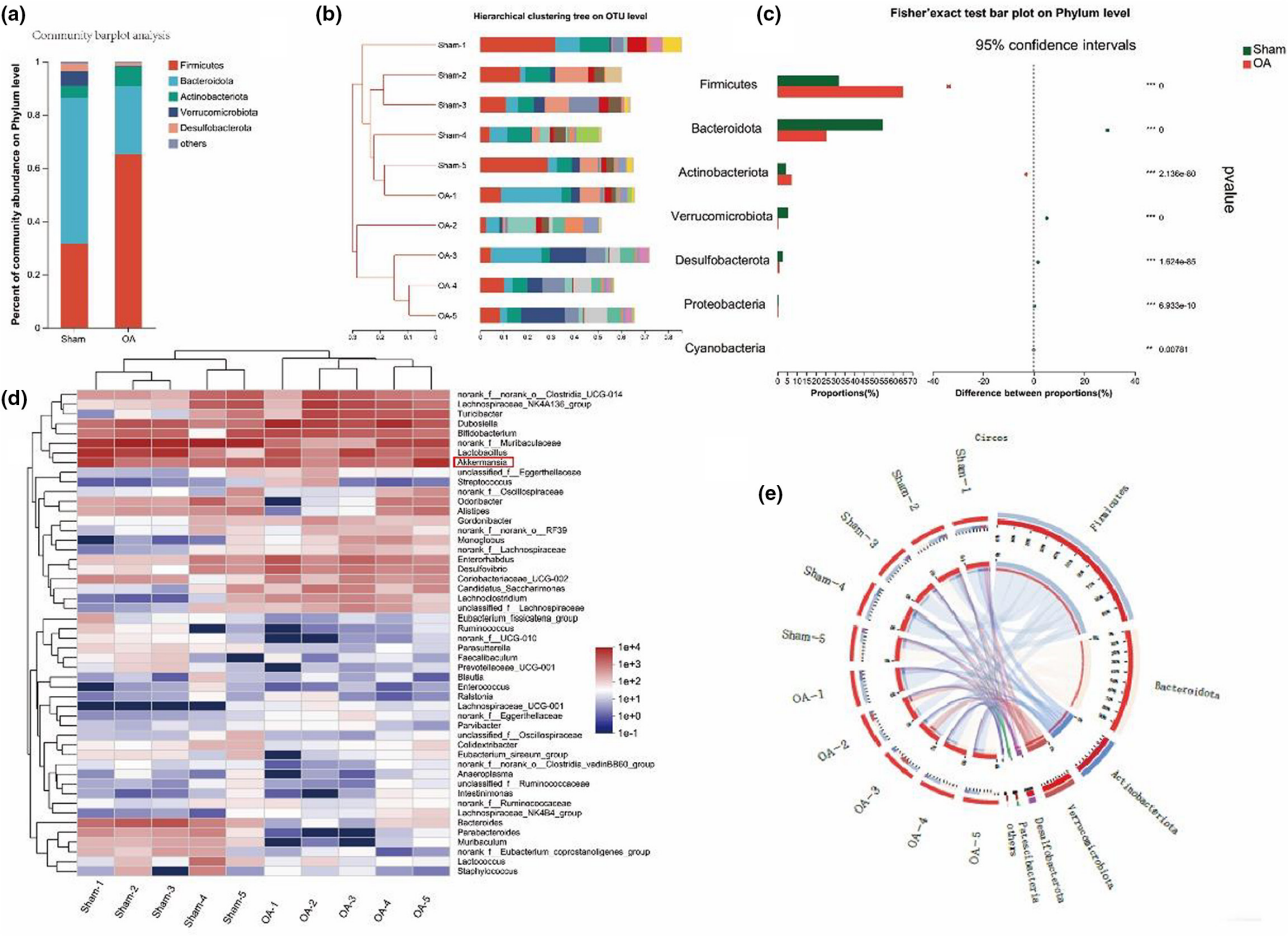
OA‐induced changes in the gut microbiota. (a) Relative bacterial abundance at the phyla level in the cecum of mice.(b) Hierarchical clustering tree on OUT level. (c) Fisher's exact test bar plot on phylum level. (d,e) Heat map and circus analysis of gut microbiota. OA, osteoarthritis; AUC, area under the curve; *p < 0.05; **p < 0.01; ***p < 0.001.

First, the changes in gut flora in osteoarthritis were explored by 16S rRNA gene sequencing. We found that the OA group showed a significant increase in Firmicute, Actinobacteriota while Bacteroidota, Verrucomicrobiota, Desulfobacterota, Proteobacteria, Cyanobacteria showed significant downregulation than the sham group (Figure 3). In addition, analysis of changes in the composition of the gut microbiota through hierarchical clustering tree. These results further suggest that there are significant differences in the composition of the gut microbiota between osteoarthritic and normal mice.

**FIGURE 4:**
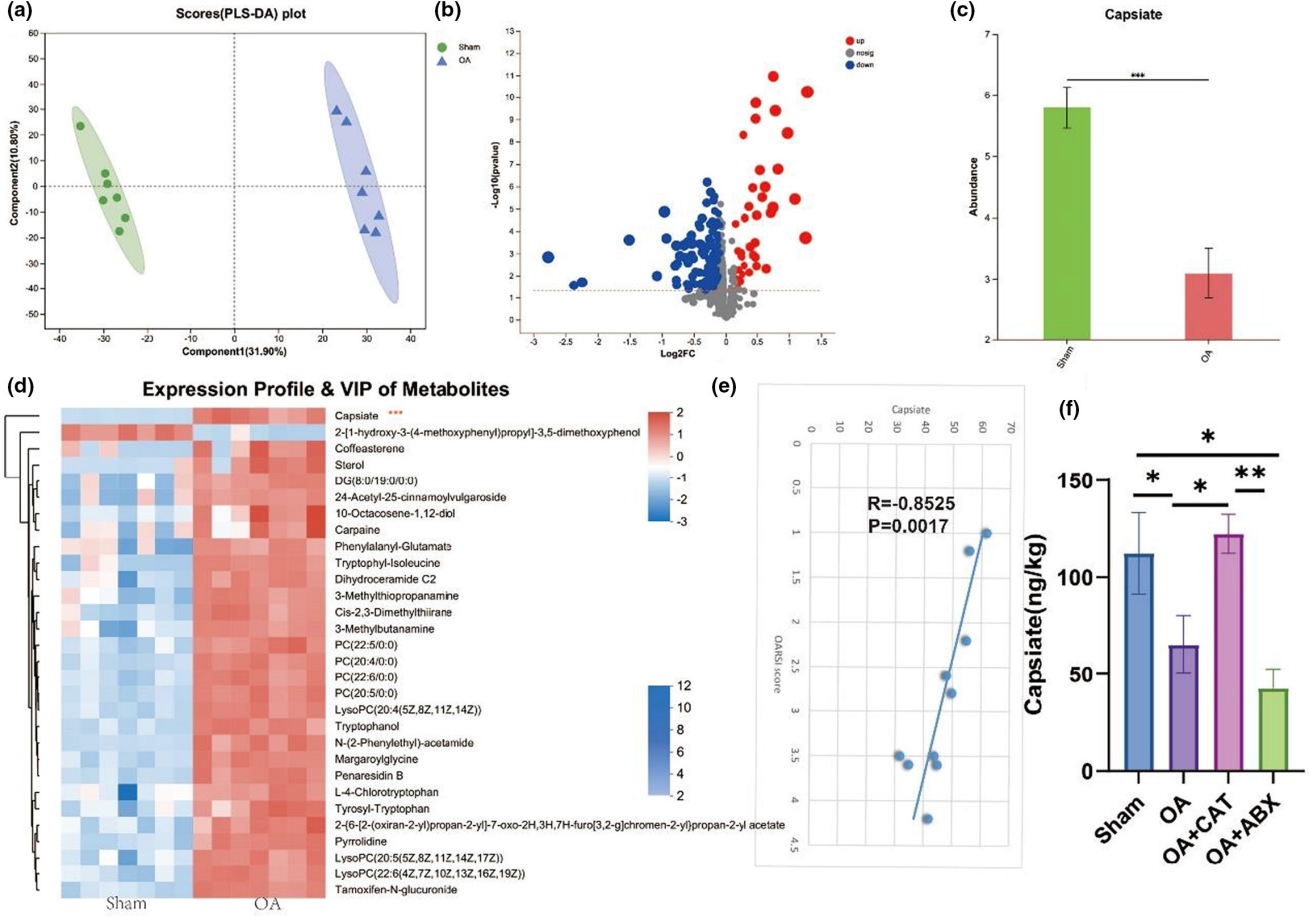
OA‐induced changes in the gut microbiota‐related metabolites. (a) Scatter plots of PLS‐DA analysis of metabolomics of cecal content. (b) Volcano plot showing the differentially accumulated [log2 (fold change) on *X*‐axis] and significantly changed [−log10 (*p*) on *Y*‐axis] metabolites in the Sham and OA group. (c) CAT levels in OA (nontargeted). (d) Heat map of gut microbiota‐related metabolites. (e) The correlation analysis between CAT and ORASI score. (f) CAT levels after ABX treatment in OA mice. OA, osteoarthritis; CAT, Capsiate; AUC, area under the curve; *p < 0.05; **p < 0.01; ***p < 0.001.

As shown in Figure 4, we next further analyzed the role of gut microbiota metabolites in osteoarthritis using flora untargeted metabolomics. PLS‐DA showed a different metabolite profile between the two groups. The composition of the thermogram and volcano plot analysis revealed a significant decrease in CAT relative to the healthy group in the OA group, with statistical differences. The negative correlation between OARSI score and CAT was investigated which may provide that CAT, a gut microbiota metabolite, is a potentially reliable drug for the treatment of osteoarthritis.

And then we analyzed the changes in CAT of gut microbiota metabolites in mice with osteoarthritis after the depletion of gut microbiota using antibiotics. The results showed a significant decrease in CAT levels after the depletion of gut microbiota in osteoarthritic mice. Therefore, our results show that gut microbiota in osteoarthritic mice can produce CAT which may be related to the development of OA.

### 3.5 CAT inhibits osteoarthritis‐induced ferroptosis

**FIGURE 5:**
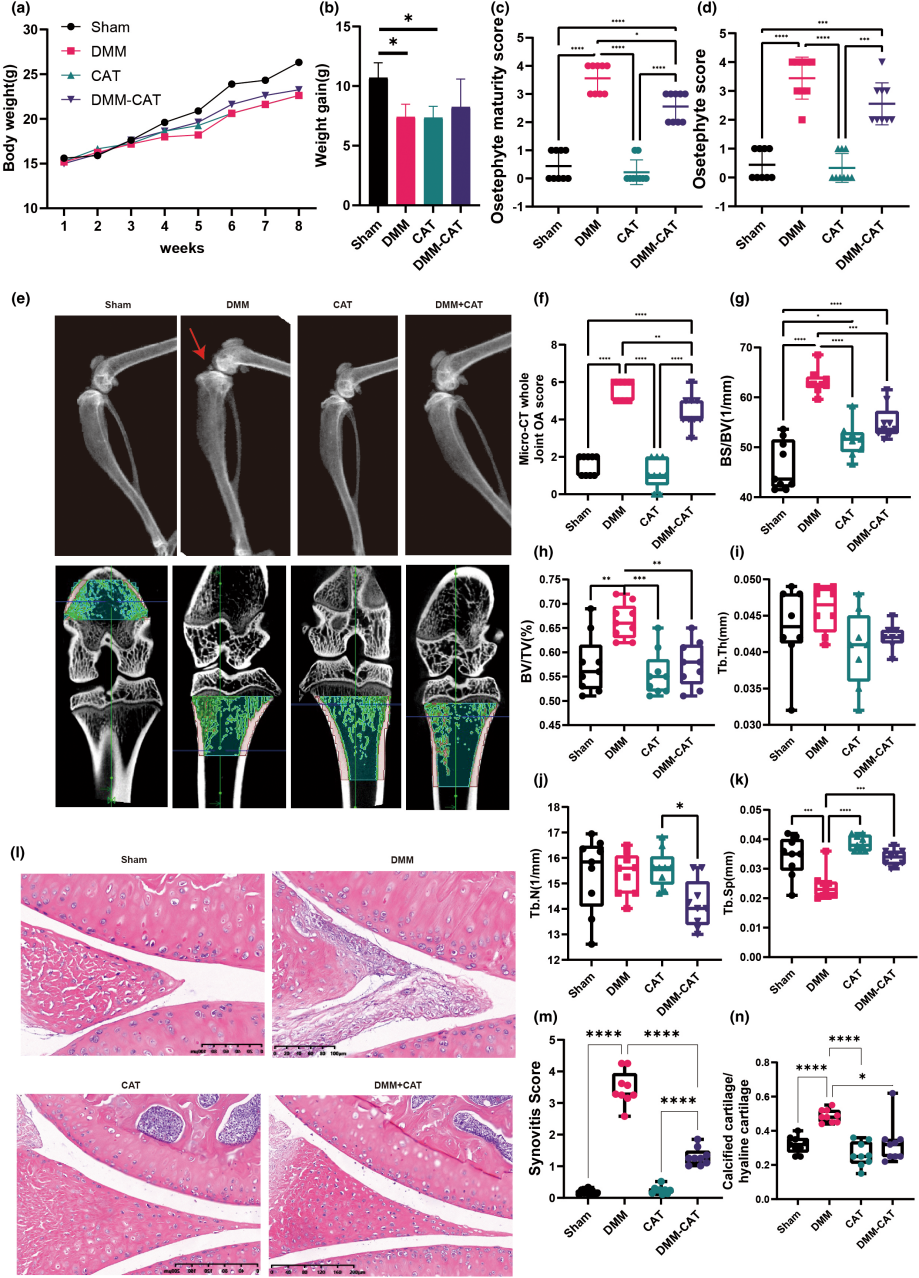
CAT changes the body weight gain and improved the development of OA mice. (a) The change of body weight. (b) body weight gain. (c) Osteophyte score. (d) Osteophyte maturity score. (e) Representative diagram of DXA. (f) Micro‐CT whole joint OA score. (g) BS/TV in tibia. (h) BV/TV in tibia. (i) Tb.Th in tibia. (j) Tb.N in tibia. (k) Tb.Sp in tibia. (l) Representative figure of HE staining. (m) Synovitis in tibia. (n) calcified cartilage/hyaline cartilage in tibia. All data are from *n* = 9 independent experiments. OA, osteoarthritis; CAT, Capsiate; AUC, area under the curve; GSH, glutathione; GSH/GSSH, glutathione/oxidized glutathione; DXA, dual energy x‐ray absorptiometry; BV/TV, bone volume over total volume; Tb.Th, trabecular thickness; Tb.N, trabecular number; Tb.Sp, trabecular spacing; OARSI; Osteoarthritis Research Society International. *p < 0.05; **p < 0.01; ***p < 0.001.

Body weight growth differed statistically between the Sham and DMM + CAT groups. As shown in Figure 5. The CAT group and the DMM with CAT group were considerably improved in the DMM group's osteophyte score and osteophyte maturity score. The CAT intervention had a considerable influence on the microstructure of the subchondral bone in osteoarthritic mice. The CAT group had a lower joint OA score and Tb.Sp, but a higher BV/TV and BS/BV than the Sham group. Meanwhile, the DMM‐CAT group had a worse joint OA score, BV/TV, and BS/BV than the DMM group. HE staining results showed synovial lesions and changes in cartilage thickness, and the results showed reduced synovial scores and the ratio of calcified cartilage to hyaline cartilage with CAT. In summary, CAT improved the development of osteoarthritic mice.

**FIGURE 6:**
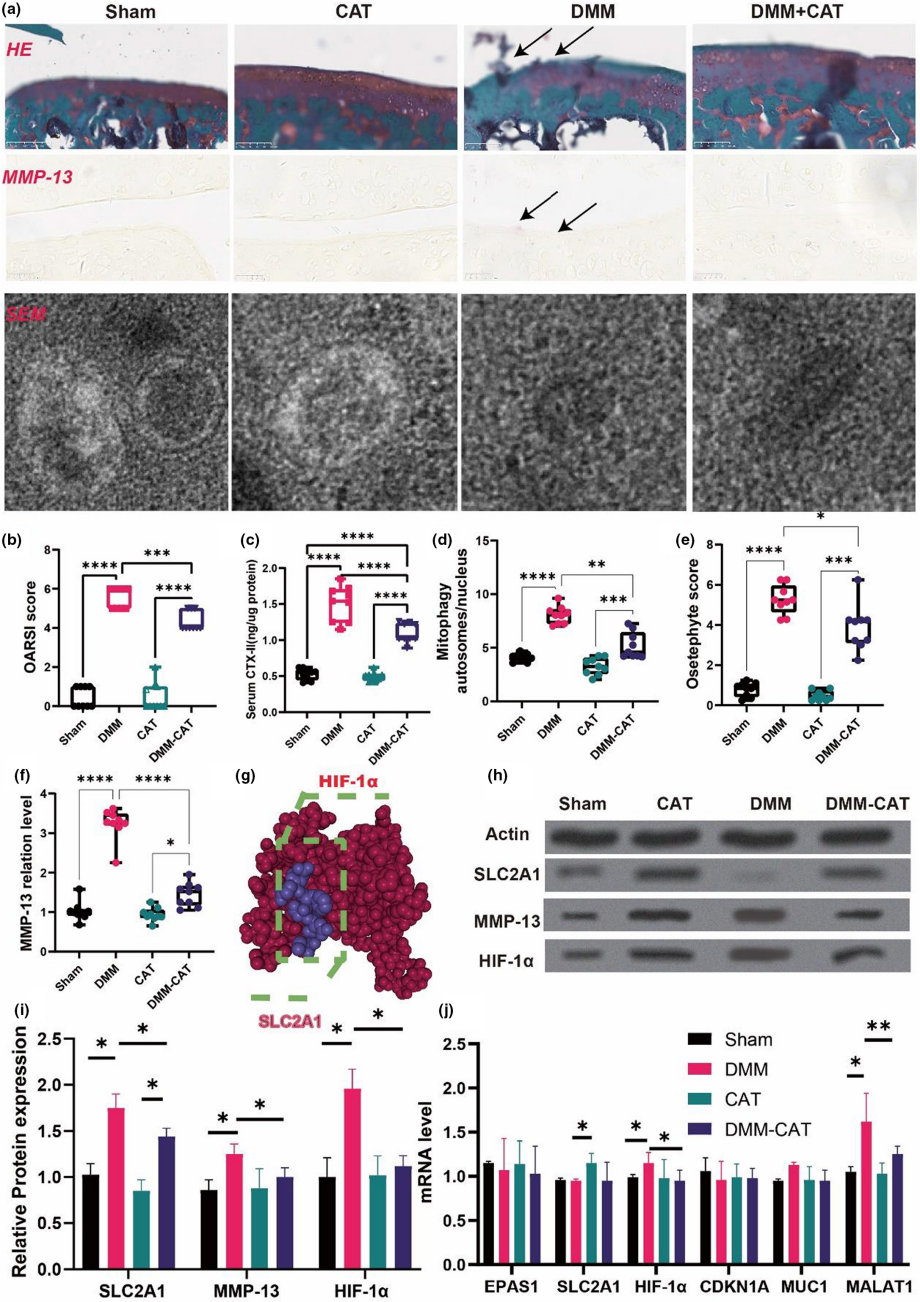
CAT regulated the SLC2A1, MMP‐13, HIF‐1α expression and improved the development of OA mice. (a) Representative diagram of Safranin‐O/Fast Green staining, immunofluorescence, scanning electron microscope. (b) OARSI score. (c) serum CTX‐II. (d) Mitophagy autosomes/nucleus. (e) Osteophyte score. (f) MMP‐13 protein level. (g) 3D binding structure of HIF1 and SCL2A1 determined via molecular modeling and docking studies. (h) Western blot. (i) Relative protein expression. (j) mRNA level. All data are from *n* = 9 independent experiments. OA, osteoarthritis; CAT, Capsiate; AUC, Area Under The Curve; GSH, glutathione; GSH/GSSH, glutathione/oxidized glutathione; DXA, dual energy x‐ray absorptiometry; BV/TV, bone volume over total volume; Tb.Th, trabecular thickness; Tb.N, trabecular number; Tb.Sp, trabecular spacing; OARSI, Osteoarthritis Research Society International. MMP‐13, matrix metalloproteinase‐13; DMM, destabilized medial meniscus;**p < 0.01; ***p < 0.001.

The assessment of cartilage damage in the joint using Safranin‐O/Fast Green staining and OARSI score, as shown in Figure 6a,b, demonstrated the influence of CAT on cartilage damage and identified a substantial increase in OARSI scores, mitophagy autosomes/nucleus, osteophyte score, MMP‐13 protein level in the DMM compared to the Sham group. The CAT and DMM‐CAT groups likewise had significantly lower OARSI ratings than the Sham group. Furthermore, blood CTX‐II levels were considerably greater in the DMM group than in the Sham group, but reduced in the DMM‐CAT group. According to the findings, CAT ameliorates the progression of osteoarthritis and inhibits the level of mitochondrial autophagy.

### 3.6 SLC2A1 regulated HIF‐1α level and oxidative stress level by inhibitor Ferroptosis

Previous bioinformatics investigation revealed that the SLC2A1 and HIF‐1α signaling pathways play a crucial role in ferroptosis‐related OA. Furthermore, to identify the specific binding sites between the two proteins, we constructed a model of the proposed SLC2A1: HIF‐1α by molecular modeling and docking (Figure 6g). Based on our docking and protein–protein interface analyses, we found that the vina score between SLC2A1 and HIF‐1α is −5.5, and the cavity size is 466. Thus, the results of mRNA and protein suggested the potential role of SLC2A1 and HIF‐1α in CAT‐induced ferroptosis‐inhibited OA.

Consequently, we initially detected protein expression levels in chondrocytes and discovered that the DMM group had considerably higher SLC2A1, MMP‐13, and HIF‐1α protein levels than the Sham group. The DMM‐CAT group, on the other hand, drastically reduced the levels of SLC2A1, MMP‐13, and HIF‐1α proteins in the DMM group. The DMM group had lower levels of HIF‐1α and MALAT1 mRNA than the Sham group. Meanwhile, the CAT group rose much more than the DMM group in SLC2A1. Furthermore, the DMM‐CAT group had lower levels of HIF‐1α and MALAT1 than the DMM group.

### 3.7 CAT‐decreased oxidative stress by SLC2A1 and HIF‐1α may be associated with ferroptosis

**FIGURE 7:**
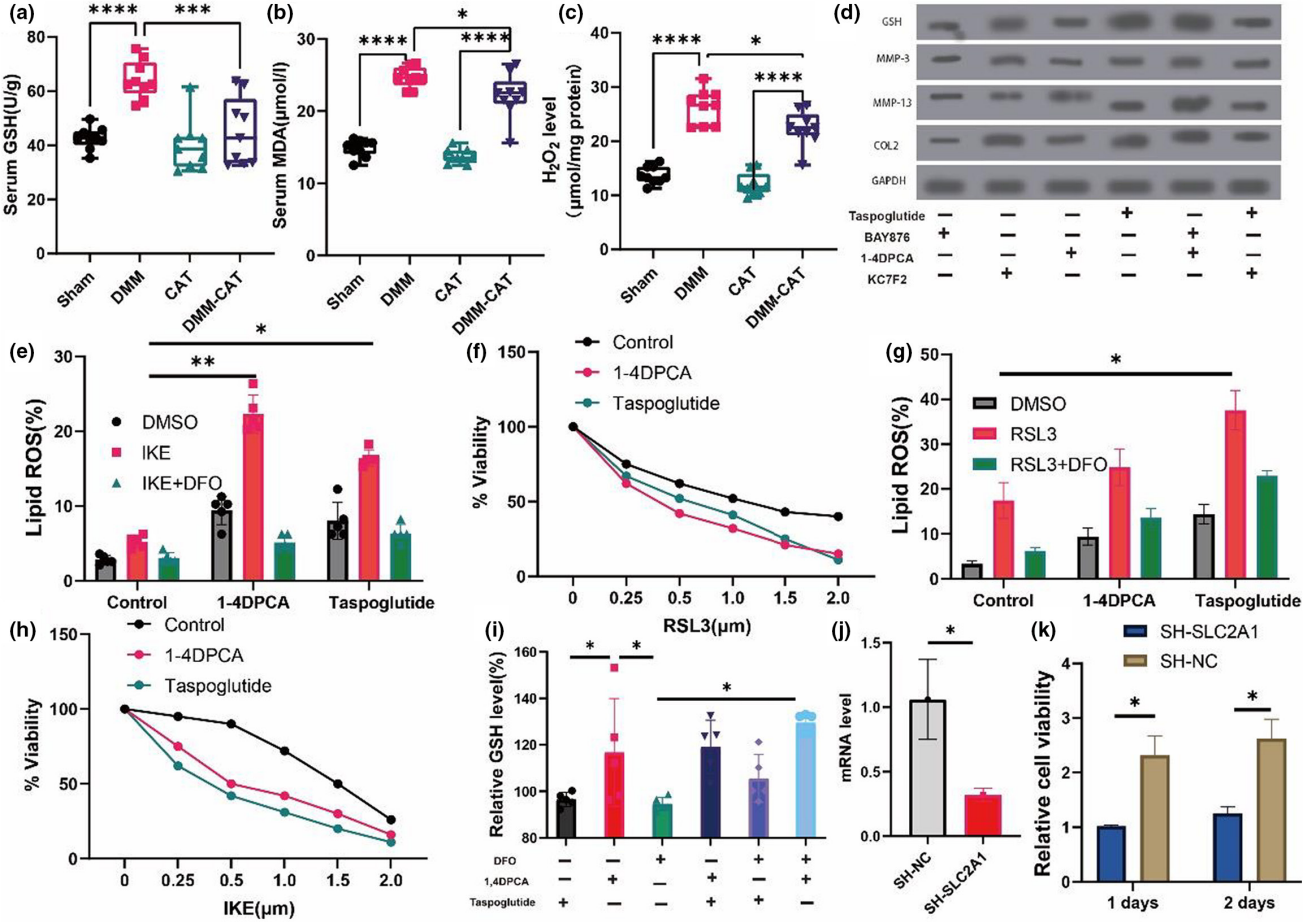
CAT‐related the oxidative stress in OA mice and SLC1A3‐HIF1α‐MALAT1 axis in cell experiment. (a) Serum GSH. (b) Serum MDA. (c) Serum H_2_O_2_. (d) Western blotting analysis by treated with SLC2A1, 1‐4DPCA and KC7F2. (e) Cells were treated as indicated for 18 h (IKE, 1 μM) in the presence of the CAT (1 μM) and lipid ROS accumulation was measured by BODIPY C11 staining coupled with flow cytometry. (f) Relative viability of fibroblasts derived and primed with control, 1‐4DPCA and SLC1A3 (20 ng/mL) for 72 h, followed by treatment with different concentrations of IKE. (g) Cells were treated as indicated for 18 h (RSL3, 1 μM) in the presence of the CAT (1 μM) and lipid ROS accumulation was measured by BODIPY C11 staining coupled with flow cytometry. (h) Relative viability of fibroblasts derived and primed with control, 1‐4DPCA and SLC1A3 (20 ng/mL) for 72 h, followed by treatment with different concentrations of RSL3. (i) relative GSH level treated by CAT, IKE, and RSL3. (j) SLC2A1 mRNA level in sh‐SLC2A1 experiment. (k) The 1 days and 2 days of cell viability in sh‐SLC2A1 experiment. (f) flow cytometry in sh‐SLC2A1 experiment. All data are from *n* = 9 independent experiments. HB, hemoglobin; MAO, monoamine oxidase; OA, osteoarthritis; AUC, area under the curve; GSH, glutathione; GSH/GSSH, glutathione/oxidized glutathione; OARSI; Osteoarthritis Research Society International. *p < 0.05; **p < 0.01; ***p < 0.001.

The oxidative stress level in OA mice was also altered by CAT. Serum GSH, MDA, and H_2_O_2_ levels were considerably higher in the DMM group than in the Sham group, whereas the DMM‐CAT group had significantly lower GSH, MDA, and H_2_O_2_ levels than the DMM group (Figure 7a–c). We discovered that CAT treatment with OA is related to elevated SLC2A1 levels and decreased HIF‐1α expression and oxidative stress levels.

We further analyzed the changes in protein levels associated with HIF‐1α and SLC2A1 agonists and inhibitors on chondrocytes after the intervention. As shown in Figure 7d, HIF‐1α agonists 1,4DPCA and SLC2A1 agonists taspoglutide increased remarkedly the level of GSH, MMP‐3, MMP‐13, and COL2 but HIF‐1α inhibiter KC7FC and SLC2A1 inhibiter BAY876 significantly decreased GSH, MMP‐3, MMP‐13. In addition, GSH, MMP3, MMP‐13, and COL2 levels remained increased with the simultaneous use of HIF‐1α agonists and SLC2A1 inhibitors, but the increase in this protein level was reversed with the treatment with HIF‐1α inhibitors and SLC2A1 agonists. The above results may suggest that SLC2A1 affects chondrocytes through HIF‐1α in CAT treatment with the development of OA.

Further confirmation of the relationship between ferroptosis and SLC2A1 and HIF‐1α. Due to lipid peroxidation being an important part of ferroptosis, we used primary chondrocytes from osteoarthritis patients to analyze the role of SLC2A1 and HIF‐1α in the progression of osteoarthritis. The intervention of SLC2A1 and HIF‐1α using SLC2A1 agonists taspoglutide and HIF‐1α agonists 1,4DPCA, and associated lipid peroxidation induced by both IKE and RSL3. Interestingly, it is found that SLC2A1 and HIF‐1α agonists inhibited the chondrocyte viability with different concentrations of RSL3 and IKE. In addition, SLC2A1 and HIF‐1α effectively sensitized chondrocytes to RSL3 and IKE‐induced lipid ROS levels (Figure 7e–h).

Finally, we analyzed the effect of CAT on HIF‐1α and SLC2A1 activation and showed that HIF‐1α activation would increase GSH levels significantly more than SLC2A1 activation, but HIF‐1α activation could still increase GSH levels when ferroptosis inhibitors were also used (Figure 7i). The above study further confirmed that ferroptosis may be ameliorated by SLC2A1‐HIF‐1α.

### 3.8 Knockout SLC2A1 increases HIF‐1α and MALAT1 in CAT treatment‐chondrocytes cells

To further understand the role of SLC2A1 in downstream HIF‐1α and MALAT1, we knocked out SLC2A1 and confirmed that the knockout was effective by detecting SLC2A1 mRNA expression in CAT treatment‐chondrocytes cells (Figure 7j). Cell activity was considerably reduced 1 and 2 days after the SLC2A1 knockdown (Figure 7k).

**FIGURE 8:**
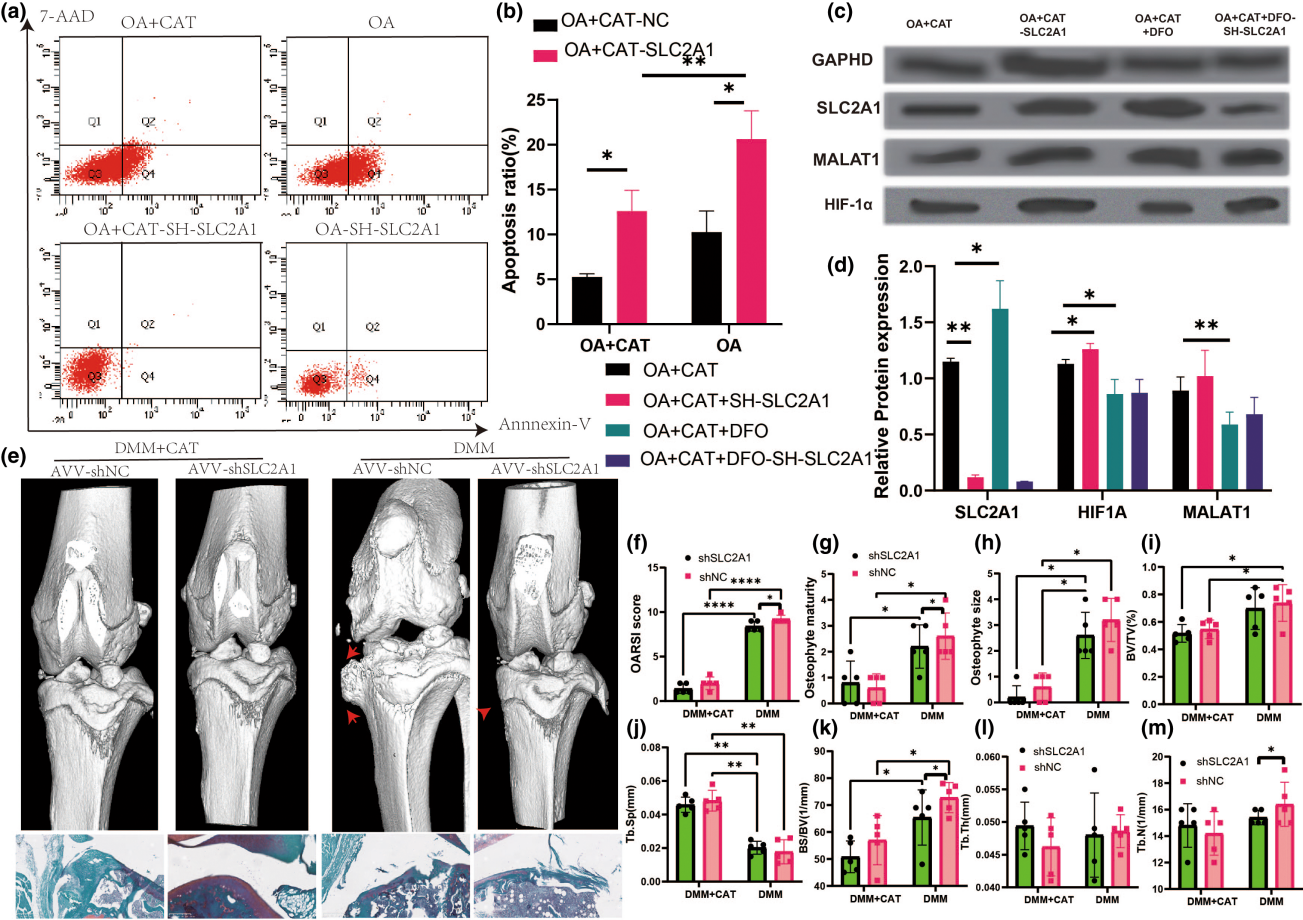
Apoptosis and protein level in sh‐SLC2A1 experiment and SLC2A1 downregulation improve CAT treatment with OA progression. (a) flow cytometry in sh‐SLC2A1 experiment. (b) Apoptosis in sh‐SLC2A1 experiment. (c) Western blot in sh‐SLC2A1 experiment. (d) Relative protein expression in sh‐SLC2A1 experiment. (e) Three‐dimensional models of mice knee joints were injected intra‐articularly with AAV carrying SLC2A1‐specific shRNA and analyzed 8 weeks after surgery. (f) OARSI score. (g) Osteophyte maturity. (h) Osteophyte size. (i) BV/TV in tibia. (j) Tb.Sp in tibia. (k) BS/TV in tibia. (l) Tb.Th in tibia. (m) Tb.N in tibia. All data are from *n* = 9 independent experiments. OA, osteoarthritis; CAT, Capsiate; AUC, Area Under The Curve; GSH, glutathione; GSH/GSSH, glutathione/oxidized glutathione; DXA, dual‐energy x‐ray absorptiometry; BV/TV, bone volume over total volume; Tb.Th, trabecular thickness; Tb.N, trabecular number; Tb.Sp, trabecular spacing; OARSI, Osteoarthritis Research Society International. MMP‐13, matrix metalloproteinase‐13; DMM, destabilized medial meniscus;**p < 0.01; ***p < 0.001.

Flow cytometry examination of apoptosis revealed that SLC2A1 knocking down dramatically raised the amount of autophagy in CAT treatment‐chondrocytes cells (Figure 8a,b). Furthermore, we supplemented CAT in SLC2A1 knockdown chondrocytes and discovered that knocking down SLC2A1 followed by CAT supplementation did not influence the levels of SLC2A1, HIF‐1α, and MALAT1. SLC2A1 knockdown also showed no influence on HIF‐1α or MALAT1 levels (Figure 8c,d). In conclusion, we confirmed that SLC2A1‐ HIF‐1α may play an essential role in CAT‐mediated ferroptosis in OA by knocking down SLC2A1.

### 3.9 SLC2A1 modulated pathogenesis of CAT treatment with OA

Finally, to clarify the effect of SLC2A1 on the onset and progression of osteoarthritis treatment with CAT, 8‐week‐old mice were injected intra‐articularly with AVV‐specific expression of SLC2A1‐specific shRNA. qPCR results showed that SLC2A1 silencing was successful. Radiographic and pathological examinations were performed after 2 weeks of injection. In contrast to the DMM + CAT group, injected with SLC2A1‐specific shRNA had no significant effect on joint degeneration, but for the DMM surgery group, intra‐articular injection of SLC2A1‐specific shRNA significantly reduced the OARSI score and osteophyte maturity score. Micro‐CT results showed that BS/BV and Tb.N in subchondral bone microstructure that was significantly reduced in SLC2A1 knockdown with the DMM group but not significantly different in the DMM + CAT group. Therefore, this part further confirms that SLC2A1 modulated the pathogenesis of CAT treatment with OA (Figure 8e–p, Figure S4).

## 4 DISCUSSION

Osteoarthritis (OA) is a severely debilitating chronic disease characterized by progressive tissue remodeling and loss of joint function and is the most prevalent disease of the synovial joints, with aging being the major risk factor for OA (Quicke et al., 2022; Yue & Berman, 2022). The inflammatory and catabolic mediator changes present during the onset of OA are consistent with those found in the “classic” senescent cellular pathway (McCulloch et al., 2017). There are data suggesting age‐related changes in joint cells and tissues, including cellular senescence, ferroptosis, autophagy, epigenetic alterations, and matrix remodeling, which contribute to the development and progression of osteoarthritis (Copp et al., 2022; Varela‐Eirin et al., 2018). In our study, we identified for the first time the gut microbiota metabolite CAT in osteoarthritic mice and also found a significant negative correlation between OARSI scores and CAT levels. These suggest that the gut microbiota metabolite CAT is a potentially reliable drug for the treatment of osteoarthritis. In addition, we found the role of ferroptosis in osteoarthritis. Also, inhibition of ferroptosis reduced cartilage damage in vivo and in vitro, and activation of ferroptosis promoted cartilage damage, which may be related to the inhibition of chondrocyte senescence. Also, we found that CAT inhibited HIF‐1a expression and reduced ferroptosis‐dependent osteoarthritis progression by activating SLC2A1. In summary, CAT, a metabolite of gut microbiota, is a potential drug for the treatment of ferroptosis‐relative osteoarthritis and the association between the ‘gut‐joint’ axis may provide a new strategy for the treatment of aging diseases.

The current work explored the relationship between iron hemostasis and OA and its mechanism. The pathophysiology of OA is complicated and involves several elements including validation, mechanical signaling, inter‐organ connections, cell death, and oxidative stress (Cicuttini & Wluka, 2014; Goldring & Goldring, 2016; Grandi & Bhutani, 2020; Guan et al., 2020; Guan, Luo, et al., 2022; Loughlin, 2022; Wen & Lohmander, 2014). Since the role of iron homeostasis in OA cannot be fully simulated using animal or cellular models, we first examined the effect of iron homeostasis on OA using clinical data and discovered iron overload in osteoarthritic patients, which was validated by the LASSO prediction model. Furthermore, as the aging process advances, iron accumulates in many tissues and organs and the iron level also rises with age, which is consistent with our findings (Mangan, 2021). Iron overload would promote the expression of chondrocytes catabolic markers, MMP3 and MMP13 expression in previous studies (Jing et al., 2021). Qu et al found that transferrin saturation had a positive causal association with osteoarthritis (Xu et al., 2022). In a Mendelian randomization study, serum iron plays a causal role in OA in women (Qu et al., 2021). In our study, patients with OA exhibited higher serum iron levels, but lower transferrin and total iron binding capacity levels. This study might lead to the discovery of novel biological markers of iron homeostasis‐related markers in OA patients.

In addition, the relationship between Iron and ferroptosis is currently unknown (Doll & Conrad, 2017). Among these forms of nonapoptotic cell death, ferroptosis (derived from the Greek word ptosis, meaning “a fall,” and ferrum, the Latin word for iron) has been first described in 2012 as an iron‐dependent form of RCD, which is characterized by the requirement of redox active iron (Dixon et al., 2012). At the heart of ferroptosis is the selenoenzyme glutathione peroxidase 4 (Gpx4), which for its unique activity to prevent uncontrolled peroxidation of phospholipids (PLs) has been proposed to be the most central downstream ferroptosis regulator (Friedmann Angeli et al., 2014). While a certain amount of iron is essential for cell proliferation and survival, iron accumulation along with an increase in lipid‐associated radicals and lipid peroxides, both hallmarks of ferroptosis, may trigger cell death (Doll & Conrad, 2017). The significant positive correlation found between iron overload and osteoarthritis in our study may also provide a theoretical basis to explain the role of ferroptosis in osteoarthritis.

Capsiate, first isolated from “CH‐19 bell pepper”, is a capsaicin analog. CAT enhances energy metabolism mainly by activating the SNS. Chili pepper intake (CI) over 2 weeks significantly reduced body weight, body fat percentage, and abdominal fat in human and rodent models. Therefore, CAT are considered as a possible dietary supplement for improving obesity (Hwang et al., 2021; Kwon et al., 2013). CAT has a significant effect on both the metabolic and secretory states of the body (Gupta et al., 2022). Previous studies have found that CAT inhibits the absorption of fatty acids in the intestine and increases insulin and glycogen levels in rats to reduce blood glucose levels (Kwon et al., 2013). CAT also improves energy expenditure and metabolism, inhibits body fat accumulation, and has antioxidant, anti‐inflammatory, and anti‐tumor activities (Hwang et al., 2021; Zang et al., 2018; Zsiborás et al., 2018). Deng et al. (2021) found that the gut microbiota metabolite capsiate can activate ferroptosis in intestinal ischemia reperfusion. Capsiate‐pretreated human keratinocytes inhibited intracellular reactive oxygen species (ROS), which activate the mitogen‐activated protein kinase and nuclear factor‐kappaB (NF‐kappaB) pathways (Lee et al., 2010). Capsiate may protect the skin from UVB‐induced adverse effects and these results provide a molecular basis for understanding its effects on inflammation and angiogenesis (Deng et al., 2021; Yamasaki et al., 2010; Zunun‐Perez et al., 2017). However, the role and mechanism of CAT in osteoarthritis are not clear. Our study is the first to analyze CAT as a metabolite of gut microbiota in mice with osteoarthritis, and in addition, CAT inhibited HIF‐1a expression and reduced ferroptosis‐dependent osteoarthritis progression by activating SLC2A1, which provides an important strategy for CAT as a potential drug for the treatment of osteoarthritis.

SLC2A1, a major regulator of ferroptosis, has been linked to several diseases, including intracerebral hemorrhage, Alzheimer's disease, and colorectal cancer (Liu, Li, et al., 2021; Liu, Yang, et al., 2022; Wang et al., 2022). SLC2A1 modulates glucose transporters and allows human chondrocytes to adjust to varying extracellular glucose concentrations (Rufino et al., 2013). In complement to the anticipated ferroptosis, we discovered a previously unknown involvement for SLC2A1 in cartilage metabolism. SLC2A1 knockdown during CAT treatment with OA might be via the HIF‐1α signaling pathway. In a previous study, the expression of SLC2A1 was increased, whereas HIF‐1αwas decreased during osteoclastogenesis, which inhibits the bone‐resorbing function of osteoclasts (Indo et al., 2013). Ren et al discovered that OA responds to low oxidative stress in chondrocytes by changing the expression of oxygen‐regulated genes and that hypoxia regulation of facilitated SLC2A1 was mediated by HIF‐1α (Ren et al., 2008). These findings are consistent with our conclusion that sh‐SLC2A1 could regulate the expression of HIF‐1α in CAT treatment with OA mice (Ancey et al., 2021).

HIF‐1α regulates adaptive responses to hypoxia conditions and mediated mitophagy has a protective role in several diseases (Gonzalez et al., 2018; Prabhakar et al., 2020). HIF‐1 α was detected in the nuclear extracts of chondrocytes which were isolated from normal or OA cartilages and cultivated under normoxic condition (Hu et al., 2020). HIF‐1α conditional KO led to massive chondrocyte cell death in the growth plate (Schipani et al., 2001). Chu et al. (2014) found that HIF‐1a levels in the serum and synovium were closely related to the radiographic severity of OA and may serve as an alternative biomarker for the progression and prognosis of knee OA. In our study, we also found a significant increase in HIF‐1 α in the cartilage of mice with osteoarthritis, and the expression of HIF‐1 α was significantly inhibited by the use of ferroptosis inhibitors.

Ferroptosis is a form of programmed cell death distinct from apoptosis, necrosis, pyroptosis, and autophagy (Liang et al., 2022). Ferroptosis is an iron‐dependent process of phospholipid peroxidation that leads to cell membrane damage and induces cell death. What's more, iron accumulation in senescent cells is coupled with impaired ferritinophagy and inhibition of ferroptosis (Wang et al., 2021). Impaired ferritin degradation explains the iron accumulation phenotype of senescent cells, whereby iron is effectively trapped in ferritin creating a perceived cellular deficiency. Accordingly, senescent cells were highly resistant to ferroptosis (Masaldan et al., 2018). Miao et al. (2022) found that inhibition of ferroptosis through modulation of GPX4 and extracellular matrix improved osteoarthritis progression. In line with this, Zhou et al. (2021) found that inhibition of ferroptosis reduced osteoarthritis leading to cartilage damage. In our study, we analyzed for the first time the link between the osteoarthritic intestinal metabolite CAT and ferroptosis, providing a new mechanism by which CAT protects against osteoarthritic progression, and in addition, we analyzed the possible mechanisms of gut microbiota and metabolites on osteoarthritis‐induced ferroptosis.

## 5 LIMITATION

The present study also has some limitations. The present study was analyzed using gut microbiota and metabolomics approaches, and although the CAT found was significantly variable and strongly associated with OA, it is not clear which specific gut microbiota are associated with CAT production. This is important to further unravel the relationship between gut microbiota and CAT. Secondly, SLC2A1 was studied in this study using adenoviral interference, which needs to be further studied in future studies using knockout animals.

## 6 CONCLUSION

Lastly, our work incorporated the findings of clinical, animal, and cellular level investigations to provides conclusive evidence of the association between iron homeostasis and OA. Our findings indicated that CAT as a metabolite of intestinal microorganisms exhibits better osteoarthritic protective effects. To our knowledge, we are the first to report that the protective effect of CAT against osteoarthritis is mediated by the inhibition of HIF‐1 α and activation of SLC2A1, thereby inhibiting ferroptosis progression. This study provides new ideas and potential drugs for the treatment of osteoarthritis and provide new ideas for possible etiologies of aging diseases.

## AUTHOR CONTRIBUTIONS

Conception and design: ZYG, LYL, ZQG. Acquisition, analysis, and interpretation of the data: JX, SFL,ZYG, KY. Drafting and writing: ZYG. Final approval of the article: ZYG, SFL, JX, LYL, ZQG, KT.

## FUNDING INFORMATION

This work was supported by grants from the Clinical Research Project of Shanghai Tenth People's Hospital (YNCR2C027) and Research Fund of Shanghai Tongren Hospital, Shanghai Jiaotong University School of Medicine (No: TRYJ2021JC02) and Tongren Xinxing (TRKYRC‐xx202215).

## CONFLICT OF INTEREST STATEMENT

The authors declare no conflict of interest. The funders had no role in the design of the study; in the collection, analyses, or interpretation of data; in the writing of the manuscript, or in the decision to publish the results.

## Supporting information


Appendix S1

## Data Availability

The data used to support the findings of this study are included in the article.
