# Supplementary material for: The gut microbiota metabolite capsiate regulate SLC2A1 expression by targeting HIF‐1α to inhibit knee osteoarthritis‐induced ferroptosis
Source: Aging Cell. 2023 Mar 8;22(6):e13807. doi: 10.1111/acel.13807 (PMC10265160; doi:10.1111/acel.13807)
Supplement: Supplementary file 1 — Appendix S1 [file ACEL-22-e13807-s001.docx]

| Supplememnt table 1. primer list. | | |
| --- | --- | --- |
| Name | Sequence Forward | Sequence Reverse |
| siSLC2A1 | 5’-caaacucaagaagcagcuaTT-3’ | 5’-caaacucaagaagcagcuaTT-3’ |
| EPAS1 | 5’-ATAAGTTCACCCAAAACCCCAT | 5’-GGCAGCAGGTAGGACTCAAAT |
| HIF-1α | 5′-GTCACCTGGTTGCTGCAATA-3′ | 5′-CATGATGGCTCCCTTTTTCA-3′ |
| MALAT1 | 5’-GCCTGGAAGCTGAAAAACGG-3’ | 5’-TGGAAAACGCCTCAATCCCA-3’ |
| CDKN1A | 5’-CAGACCAGCCTGACAGATTTC-3’ | 5’-GGCACTTCAGGGTTTTCTCTT-3’ |
| MUC1 | 5’-CCCCAGTTGTCTGTTGGGGTC-3’ | 5’-GGATTCTACCACCACGGAGCC-3’ |
| SLC2A1 | 5’-CTTCACTGTGGTGTCGCTGT -3’ | 5’-TTCAAAGAAGGCCACAAAGC -3’ |
| Gapdh | 5’-TGC ACC ACC AAC TGC TTA G-3’ | 5’-GGA TGC AGG GAT GAT GTT C-3’ |

| Supplement Table 3. top 7 biological pathways were seleceted and shown accrding to enrichment score | | | | | | | | | | | | | |
| --- | --- | --- | --- | --- | --- | --- | --- | --- | --- | --- | --- | --- | --- |
| Term | | | | | | | Count | | % | | P-Value | | Benjamini |
| HIF-1 signaling pathway | | | | | | | 8 | | 2.35E+01 | | 2.60E-08 | | 4.60E-06 |
| Renal cell carcinoma | | | | | | | 6 | | 1.76E+01 | | 1.80E-06 | | 1.60E-04 |
| Proteoglycans in cancer | | | | | | | 7 | | 2.06E+01 | | 2.90E-05 | | 1.70E-03 |
| Pancreatic cancer | | | | | | | 5 | | 1.47E+01 | | 7.80E-05 | | 3.40E-03 |
| Bladder cancer | | | | | | | 4 | | 1.18E+01 | | 2.60E-04 | | 9.10E-03 |
| FoxO signaling pathway | | | | | | | 5 | | 1.47E+01 | | 6.30E-04 | | 1.90E-02 |
| Pathways in cancer | | | | | | | 8 | | 2.35E+01 | | 8.40E-04 | | 2.10E-02 |
| Central carbon metabolism in cancer | | | | | | | 4 | | 1.18E+01 | | 1.20E-03 | | 2.70E-02 |
| Hepatocellular carcinoma | | | | | | | 5 | | 1.47E+01 | | 1.60E-03 | | 2.90E-02 |
| Leishmaniasis | | | | | | | 4 | | 1.18E+01 | | 1.60E-03 | | 2.90E-02 |
| MAPK signaling pathway | | | | | | | 6 | | 1.76E+01 | | 1.80E-03 | | 2.90E-02 |
| MicroRNAs in cancer | | | | | | | 6 | | 1.76E+01 | | 2.20E-03 | | 3.10E-02 |
| Colorectal cancer | | | | | | | 4 | | 1.18E+01 | | 2.20E-03 | | 3.10E-02 |
| AGE-RAGE signaling pathway in diabetic complications | | | | | | | 4 | | 1.18E+01 | | 3.40E-03 | | 4.40E-02 |
| PI3K-Akt signaling pathway | | | | | | | 6 | | 1.76E+01 | | 4.00E-03 | | 4.70E-02 |
| Human T-cell leukemia virus 1 infection | | | | | | | 5 | | 1.47E+01 | | 4.40E-03 | | 4.90E-02 |
| Shigellosis | | | | | | | 5 | | 1.47E+01 | | 6.40E-03 | | 6.60E-02 |
| Ferroptosis | | | | | | | 3 | | 8.80E+00 | | 6.90E-03 | | 6.60E-02 |
| Relaxin signaling pathway | | | | | | | 4 | | 1.18E+01 | | 7.00E-03 | | 6.60E-02 |
| Gastric cancer | | | | | | | 4 | | 1.18E+01 | | 1.00E-02 | | 9.20E-02 |
| Cushing syndrome | | | | | | | 4 | | 1.18E+01 | | 1.20E-02 | | 9.80E-02 |
| Hepatitis B | | | | | | | 4 | | 1.18E+01 | | 1.30E-02 | | 1.00E-01 |
| Endometrial cancer | | | | | | | 3 | | 8.80E+00 | | 1.40E-02 | | 1.00E-01 |
| Tuberculosis | | | | | | | 4 | | 1.18E+01 | | 1.70E-02 | | 1.30E-01 |
| Neutrophil extracellular trap formation | | | | | | | 4 | | 1.18E+01 | | 2.00E-02 | | 1.30E-01 |
| Melanoma | | | | | | | 3 | | 8.80E+00 | | 2.00E-02 | | 1.30E-01 |
| Non-small cell lung cancer | | | | | | | 3 | | 8.80E+00 | | 2.00E-02 | | 1.30E-01 |
| Kaposi sarcoma-associated herpesvirus infection | | | | | | | 4 | | 1.18E+01 | | 2.10E-02 | | 1.30E-01 |
| Glioma | | | | | | | 3 | | 8.80E+00 | | 2.20E-02 | | 1.30E-01 |
| Chronic myeloid leukemia | | | | | | | 3 | | 8.80E+00 | | 2.30E-02 | | 1.30E-01 |
| EGFR tyrosine kina+F38:F40se inhibitor resistance | | | | | | | 3 | | 8.80E+00 | | 2.40E-02 | | 1.40E-01 |
| ErbB signaling pathway | | | | | | | 3 | | 8.80E+00 | | 2.80E-02 | | 1.50E-01 |
| Chemical carcinogenesis - reactive oxygen species | | | | | | | 4 | | 1.18E+01 | | 3.00E-02 | | 1.60E-01 |
| PD-L1 expression and PD-1 checkpoint pathway in cancer | | | | | | | 3 | | 8.80E+00 | | 3.00E-02 | | 1.60E-01 |
| Human cytomegalovirus infection | | | | | | | 4 | | 1.18E+01 | | 3.10E-02 | | 1.60E-01 |
| Rheumatoid arthritis | | | | | | | 3 | | 8.80E+00 | | 3.30E-02 | | 1.60E-01 |
| Coronavirus disease - COVID-19 | | | | | | | 4 | | 1.18E+01 | | 3.30E-02 | | 1.60E-01 |
| Prostate cancer | | | | | | | 3 | | 8.80E+00 | | 3.60E-02 | | 1.70E-01 |
| Endocrine resistance | | | | | | | 3 | | 8.80E+00 | | 3.60E-02 | | 1.70E-01 |
| Chagas disease | | | | | | | 3 | | 8.80E+00 | | 3.90E-02 | | 1.70E-01 |
| Parathyroid hormone synthesis, secretion and action | | | | | | | 3 | | 8.80E+00 | | 4.20E-02 | | 1.80E-01 |
| Toxoplasmosis | | | | | | | 3 | | 8.80E+00 | | 4.60E-02 | | 2.00E-01 |
| Supplement Table 4. Network analysis by STRING | | | | | | | | | | | | |  |
| node1 | node2 | coexpression | | experimentally_determined_interaction | | | | database_annotated | | automated_textmining | | combined_score |  |
| EGFR | MUC1 | 0.076 | | 0.873 | | | | 0 | | 0.989 | | 0.998 |  |
| EGFR | CD44 | 0.099 | | 0.753 | | | | 0 | | 0.989 | | 0.997 |  |
| MAPK1 | DUSP1 | 0 | | 0.837 | | | | 0.9 | | 0.776 | | 0.996 |  |
| TLR4 | CD44 | 0.095 | | 0 | | | | 0 | | 0.988 | | 0.989 |  |
| EPAS1 | VEGFA | 0.088 | | 0.316 | | | | 0.9 | | 0.696 | | 0.978 |  |
| MAPK1 | EGFR | 0 | | 0.44 | | | | 0.9 | | 0.832 | | 0.962 |  |
| EPAS1 | CA9 | 0.069 | | 0 | | | | 0.9 | | 0.606 | | 0.96 |  |
| EGFR | VEGFA | 0.089 | | 0 | | | | 0.6 | | 0.861 | | 0.944 |  |
| MAPK1 | TGFB1 | 0.049 | | 0.058 | | | | 0.9 | | 0.381 | | 0.937 |  |
| DUSP1 | ZFP36 | 0.793 | | 0.062 | | | | 0 | | 0.689 | | 0.934 |  |
| MAPK1 | NR4A1 | Supplement Table 3+F38:F40. top 7 biological pathways were seleceted and shown accrding to enrichment score | | 0.241 | | | | 0.8 | | 0.283 | | 0.881 |  |
| TGFB1 | VEGFA | 0 | | 0 | | | | 0.5 | | 0.71 | | 0.848 |  |
| EGFR | TLR4 | 0 | | 0.317 | | | | 0 | | 0.773 | | 0.838 |  |
| TGFB1 | CD44 | 0.099 | | 0 | | | | 0 | | 0.8 | | 0.812 |  |
| Supplement Table 5 miRNA and its target genes | | | | | |  |  |  |  |  |  |  |  |
| miRNA | | | Genes targeted by miRNA | | Gene count |  |  |  |  |  |  |  |  |
| hsa-miR-106a-5p | | | TGFB1, CDKN1A | | 2 |  |  |  |  |  |  |  |  |
| hsa-miR-149-3p | | | CDKN1A, CD44 | | 2 |  |  |  |  |  |  |  |  |
| hsa-miR-145-5p | | | CDKN1A, MUC1 | | 2 |  |  |  |  |  |  |  |  |
| hsa-miR-17-5p | | | CDKN1A, TGFB1 | | 2 |  |  |  |  |  |  |  |  |
| hsa-miR-4694-3p | | | TLR4, CDKN1A | | 2 |  |  |  |  |  |  |  |  |
| hsa-miR-4728-5p | | | CDKN1A, CD44 | | 2 |  |  |  |  |  |  |  |  |
| hsa-miR-6828-3p | | | CDKN1A, CD44 | | 2 |  |  |  |  |  |  |  |  |
| hsa-miR-6883-5p | | | CDKN1A, CD44 | | 2 |  |  |  |  |  |  |  |  |

**Supplementary Figure 1.** Difference genes expression in GSE98198 dataset. A. heat maps. B. volcano plots, B. Network of enriched terms., C. GSEA analysis by GSEA online tool. E enrichment analysis in GSEA.

**Supplementary Figure 2.** Network analysis between miRNA-lncRNA aixe. A. Cytoscape network visualization that were obtained with interaction scores > 0.4 according to the STRING online database. B. Cluster 2. C. Cluster 1. D. enrichment analysis of cluster 1. E. Network analysis between mRNA and miRNA. F. Network analysis between miRNA and LncRNA. G. Venn plot among three miRNA.

**Supplementary Figure 3.** UMAP2, PCA and GO enrichment analysis by 34 different expression genes with ferroptosis and GSE98918 dataset (A-F).

**Supplementary Figure 4.** Interaction difference genes expression between Ferrbo and GSE98198 and enrichment analysis by 149 miRNA with ferroptosis and GSE98918 dataset. A. Venn plot between Ferrbo and GSE98198. B-I. Enrichment analysis by GSEA online tool. J-K, enrichment analysis by 149 miRNA with ferroptosis and GSE98918 dataset.

**Supplementary Figure 5.** KEGG and GO enrichment analysis by 34 different expression genes with ferroptosis and GSE98918 dataset (A-F).

**Supplementary Figure 6.** KEGG and GO enrichment analysis by cluster 1 genes with ferroptosis and GSE98918 dataset (A-F).

**Supplementaray Figure 7** The design of animal models
